# Cognition-Linked Monocyte State Reveals Altered Myeloid–Lymphoid Coordination in Neuro-PASC

**DOI:** 10.3390/ijms27167474

**Published:** 2026-08-21

**Authors:** Barbara A. Hanson, Andrew C. Cogswell, Melissa Lopez, Janet Miller, Kristen L. Knutson, Mercedes R. Carnethon, Igor J. Koralnik

**Affiliations:** 1 Davee Department of Neurology, Northwestern University Feinberg School of Medicine, Chicago, IL 60611, USA; barbara.hanson@northwestern.edu (B.A.H.); melissa.lopez@northwestern.edu (M.L.); kristen.knutson@northwestern.edu (K.L.K.); 2 Department of Immunology-Microbiology, Northwestern University Feinberg School of Medicine, Chicago, IL 60611, USA; andrew.cogswell@northwestern.edu; 3 Northwestern Medicine, Chicago, IL 60611, USA; jmiller@nm.org; 4 Department of Preventive Medicine, Northwestern University Feinberg School of Medicine, Chicago, IL 60611, USA; carnethon@northwestern.edu

**Keywords:** post-acute sequelae of SARS-CoV-2 infection (PASC), long COVID, oxidative phosphorylation, OXPHOS, Complex V, ATP synthase, Neuro-PASC

## Abstract

Neurologic manifestations of long COVID, also called neurologic post-acute sequelae of SARS-CoV-2 infection (Neuro-PASC: NP) include persistent alteration of cognitive functions. We investigated whether these could be driven by immune perturbations. We combined flow cytometry (FC), sleep profiling, and single-cell RNA sequencing of peripheral blood immune cells from older adult (>55 years) individuals with and without NP to evaluate relationships with objective cognitive performance. NP participants showed reduced numbers of blood monocytes with increased mitochondrial superoxide, indicating an altered monocyte mitochondrial redox state. Higher peripheral capillary oxygen saturation (SpO_2_) was associated with better processing speed in NP participants. Monocyte transcriptional analyses identified mitochondrial adenosine triphosphate (ATP) synthase/Complex V (Complex V) pathway associated with cognitive performance in people without NP; this coupling was abrogated in NP patients, in whom cognitive performance instead showed an opposite relationship with Complex V. Shared leading-edge genes defined a 13-gene monocyte anchor representing this cognition-associated NP phenotype. Higher anchor scores were associated with coordinated oxidative phosphorylation and cytotoxic programs across CD3^+^ T-cell subsets in individuals without NP, but not in NP participants. T-cell receptor stratified analyses showed that this altered relationship occurred in both expanded and unexpanded T-cell populations. FC correlations also supported reduced monocyte-to-lymphocyte mitochondrial coordination in NP. These exploratory findings identify a sleep and cognition-linked monocyte mitochondrial phenotype characterized by altered myeloid–lymphoid immune coordination in NP.

## 1. Introduction

Long COVID, also termed post-acute sequelae of SARS-CoV-2 infection (PASC), remains a substantial global health problem. The cumulative global burden was estimated to have reached approximately 400 million individuals worldwide by the end of 2023, with an annual detrimental economic impact approaching $1 trillion [1]. PASC can affect multiple organ systems, and neurologic manifestations are among the most debilitating and include ‘brain fog’, cognitive dysfunction, headache, dizziness, sleep disturbance, and intense fatigue [2]. Neuro-PASC (NP) can occur after mild acute infection, and symptoms may persist for years. Age is known to influence the clinical presentation of NP, where younger and middle-aged adults are more severely affected, with few studies dedicated to older adults [3]. Characterizing the physiologic and immune processes associated with cognitive symptoms in this population is therefore necessary to identify potential mechanisms and targets for intervention.

Sleep disturbance is common in NP, but objective evaluations of sleep physiology remain relatively sparse. Polysomnography in adults with chronic insomnia after COVID-19 has demonstrated reduced sleep efficiency and prolonged wake after sleep onset [4]. More recently, adults with long COVID were found to have significantly lower average average peripheral oxygen saturation (SpO_2_) after sleep onset and during rapid eye movement (REM) than sleep-matched controls [5]. These measurements describe distinct components of sleep physiology, with mean and minimum SpO_2_ quantifying different features of oxygenation, while the apnea-hypopnea index and oxygen desaturation index measure respiratory events rather than oxygenation itself. Direct evidence linking objective nocturnal oxygenation to objective cognitive performance in PASC remains limited. A pilot study of supplemental oxygen found no overall improvement in cognitive performance, though cerebral perfusion and oxygenation have been associated with attention and daytime sleepiness after COVID-19 [6]. The relationship among objective sleep physiology, cognition, and systemic immune abnormalities which have been observed in NP is therefore poorly understood.

Mitochondrial and persistent immune abnormalities may contribute to neurologic symptoms following SARS-CoV-2 infection. During acute infection, SARS-CoV-2 exposure can promote mitochondrial reactive oxygen species (mtROS)-dependent glycolytic reprogramming in monocytes, while circulating monocytes from patients with COVID-19 pneumonia have shown impaired oxygen usage, altered mitochondrial membrane potential, and abnormal morphology [7,8]. Evidence of metabolic alteration following the acute phase is inconsistent. Bulk peripheral blood mononuclear cells (PBMCs) from individuals with PASC have demonstrated increased basal, adenosine triphosphate (ATP)-linked, maximal, and spare respiratory capacity, whereas other studies have identified increased oxygen consumption accompanied by abnormal membrane potential or impaired monocyte adaptation to oxidative stress and mitochondrial DNA depletion [9,10,11]. Our group previously found that COX7A1 in plasma, an oxidative phosphorylation (OXPHOS)-associated protein, was elevated in NP and associated with worse recovery, fatigue, subjective cognition, processing speed, and attention [12]. COX7A1 also showed an inverse relationship with other OXPHOS-associated plasma proteins in NP but a positive relationship in COVID convalescent controls (CCs). These findings support mitochondrial involvement in NP but do not identify the cellular source of the plasma proteins, establish whether this is associated with increased or decreased mitochondrial activity, or define how mitochondrial programs relate across immune cell compartments.

Studies in acute COVID-19 have identified metabolic abnormalities in myeloid and lymphoid populations measured within the same cohorts, including mitochondrial dysfunction and apoptotic susceptibility in T cells together with metabolically altered myeloid populations [13,14]. Given that mitochondrial and metabolic abnormalities have been identified in both myeloid and lymphoid populations following SARS-CoV-2 infection, it is important to consider how these compartments function in relation to one another. Myeloid and lymphoid lineages contribute complementary roles in immune activation, regulation, and resolution, yet little is known about how their metabolic programs vary together in chronic NP. We therefore combined flow cytometry (FC), objective sleep profiling, standardized cognitive testing, and single-cell RNA sequencing (scRNA-seq) to examine mitochondrial immune phenotypes in older adults with and without NP. We evaluated whether objective sleep parameters and cognitive performance were associated with cellular transcription patterns and whether the cellular phenotypes showed different relationships across groups, and finally whether these were associated with SARS-CoV-2-responsive or expanded T-cell populations.

## 2. Results

### 2.1. Study Subjects

A total of 62 participants were included in analyses using flow cytometry (FC; *n* = 19), a sleep profiler (*n* = 43), and/or single-cell RNA sequencing (scRNA-seq; *n* = 11). NP was defined as persistent neurologic symptoms following acute SARS-CoV-2 infection that lasted at least 3 months and remained active at the time of sample collection. All participants presented with mild COVID-19 during acute infection and did not require hospitalization for pneumonia or hypoxemia; this restriction was intended to limit the confounding effects of poor oxygenation and complications of severe acute disease. In the FC analysis, NP participants were compared with convalescent controls (CCs), defined as individuals with prior SARS-CoV-2 infection who did not report persistent post-acute symptoms. In the sleep profiler and scRNA-seq analyses, participants in the Study of Disparities in Sleep and Cognition in Older Adults (DISCO) with NP were designated the DISCO NP (DNP) group, whereas the non-NP subjects enrolled under the DISCO protocol (nonDNP) comparison group included both convalescent controls and individuals without documented SARS-CoV-2 infection who reported no known history of COVID-19.

Participant samples were therefore used for either FC analyses, comparing NP and CC participants, or sleep profiler/scRNA-seq analyses, comparing DNP and nonDNP participants. Groups were age- and sex-matched both within each arm and across the overall study group (Table 1).

Cognitive and quality-of-life (QoL) measures were assessed using the NIH Toolbox and the Patient-Reported Outcomes Measurement Information System (PROMIS), respectively. These measures were available for both groups in the sleep profiler/scRNA-seq analysis and for NP participants only in the FC analysis. Objective NIH Toolbox cognitive performance was similar among all participants tested, whereas the PROMIS measures showed significantly greater subjective alterations of QoL in domains of cognition, fatigue, sleep disturbance, and anxiety burden in DNP participants compared with nonDNP controls.

### 2.2. NP Is Associated with Reduced Circulating Monocytes and Increased Monocyte Mitochondrial Superoxide

**Figure 1:**
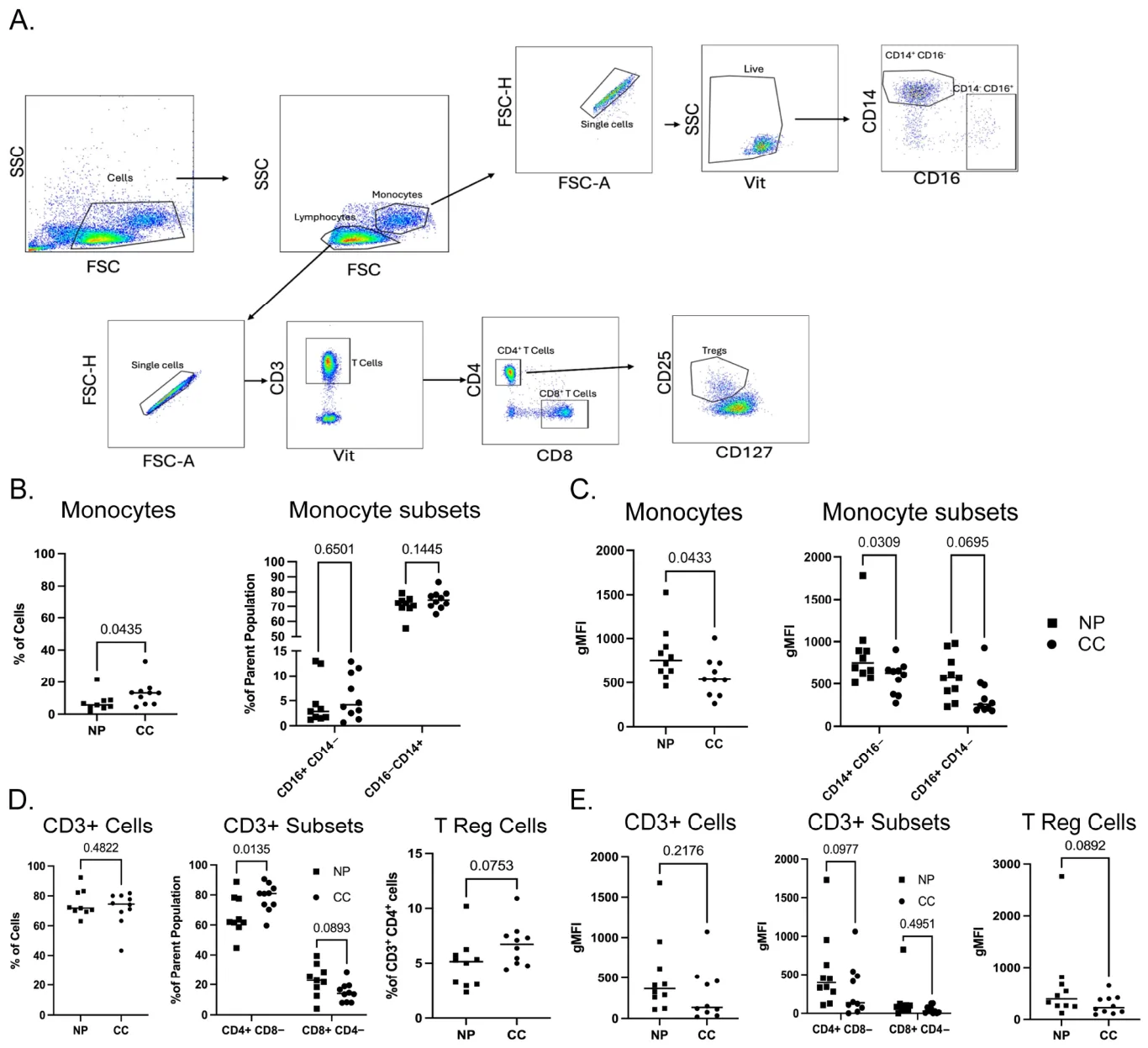
Alterations in peripheral immune cells of patients with NP. (**A**) Representative flow cytometry gating strategy for immune cell populations. Samples were first gated on the cellular population (cells), then subdivided based on size and granularity into either lymphocytes or monocytes. Both were then gated for singlet, living cells, while T cells were gated based on CD3 expression (T cells), then CD4 (CD4) and CD8 (CD8) expression, and finally CD4^+^ T cells were then gated based on CD127 and CD25 expression to identify regulatory T cells (Tregs). Monocytes were further gated based on the expression of CD14 and CD16. (**B**) Relative frequency of total monocytes (**left panel**) and monocyte subpopulations (**right panel**) based on flow gating strategy in A in NP and CC patients. (**C**) Geometric mean fluorescence intensity (gMFI) of mtROS staining in monocytes and monocyte subpopulations in NP and CC patients. (**D**) Relative frequency of total T cells (**left panel**), CD4^+^ and CD8^+^ (**middle panel**), and CD4^+^ Tregs (**right panel**) based on gating strategy in A in NP and CC patients. (**E**) gMFI of mtROS staining of total T cells (**left panel**), CD4^+^ and CD8^+^ (**middle panel**), and CD4^+^ Tregs (**right panel**) in NP and CC patients. Each dot represents an individual patient, *n* = 9 for NP patients and *n* = 10 for CC patients. Statistical tests were performed utilizing Mann–Whitney U or 2-way analysis of variance (ANOVA) where appropriate; *p* values are shown above the compared populations.

Previous studies have reported alterations in peripheral immune signatures in patients after COVID-19, including significant alterations in mitochondrial respiration [15,16]. To address whether these alterations were persistent in NP patients, flow cytometry was performed on isolated PBMCs from NP patients and CC participants to identify whether there were alterations in immune subpopulations. Immune cells were identified via the gating strategy shown in Figure 1A.

NP patients showed a significant reduction in the frequency of monocytes compared to CC participants (7.3% ± 5.9% vs. 13.1% ± 8.0%, *p* = 0.0435; Leave-one-out [LOO] NP−CC difference = −5.87% to −4.62 percentage points, *p* = 0.0085–0.0831) (Figure 1B). While the frequency of total monocytes was reduced among NP patients, there were no specific alterations to the frequency of any one subpopulation, as both the percentage of CD14^+^ CD16^−^ and the CD14^int/−^ CD16^+^ subpopulations showed no alterations compared to CC patients (Figure 1B).

We investigated whether alterations in the frequency of monocytes in NP patients could be associated with any other phenotypic variation. Increases in mitochondrial reactive oxygen species (mtROS) have been shown to lead to increased apoptosis and inflammation in monocytes and can be associated with autoimmune conditions [17,18]. Notably, active SARS-CoV-2 infection has been shown to increase mtROS in monocytes [7]. To determine whether NP patients had persistent increases in mtROS, we stained cells with MitoSOX and analyzed them via FC. We found that monocytes from patients with NP had increased mtROS (*p* = 0.0433; LOO NP−CC difference = 178.0–277.5 mean fluorescent intensity (MFI), *p* = 0.0142–0.0831) (Figure 1C). At the subpopulation level, CD14^+^ CD16^−^ monocytes had a statistically significant increase in mtROS (*p* = 0.0309; LOO NP−CC difference = 177.1–335.3 MFI, *p* = 0.0133–0.1084), while CD14^int/−^CD16^+^ monocytes showed a trend towards an increase in mtROS staining (Figure 1C). In summary, the data show that NP patients have a decreased frequency of circulating monocytes and that these monocytes show an increase in mtROS compared to monocytes from CC participants.

In parallel, we examined the phenotypes of T cells. Alterations in T cells in the acute phase of infection [19,20] and T-cell dysfunction in long COVID have been well documented [21,22]. We first examined whether there were any alterations observed in the relative frequency of T cells in NP patients compared to CC individuals. We found that while there was no statistically significant reduction in total CD3^+^ T cells compared to CC participants, there was a reduction in frequency of CD4^+^ T cells in NP patients (*p* = 0.0135; LOO NP−CC difference = −15.39 to −9.87 percentage points, *p* = 0.00069–0.0262; Figure 1D).

Given that alterations in CD4^+^ T-cell frequency are associated with increased inflammation and pathology, we next investigated if there was also a decrease in regulatory T cells (Tregs), a subset of CD4^+^ T cells which contribute to the control of inflammation. The frequency of Tregs was decreased in NP patients compared to CC individuals (4.93% ± 2.30% vs. 6.66% ± 1.93%) but this did not reach statistical significance (*p* = 0.075) (Figure 1E). Similar to the metabolic alterations in monocytes, alterations in T-cell metabolism have been widely reported in the context of COVID-19 [14,23]. In NP patients, we observed trends towards increases mtROS in CD4^+^ T cells and specifically within the Treg compartment, yet none of these reached statistical significance (Figure 1E). Taken together, these data demonstrate a decrease in the relative proportion of CD4^+^ T cells in NP patients with a trend towards a decrease in peripheral Treg cells. Furthermore, there is a trend towards an increase in mtROS in bulk CD4^+^ T cells and Treg cells.

Flow cytometry therefore identified a predominantly monocyte-altered phenotype in NP, characterized by reduced circulating monocyte frequency and increased monocyte mtROS, with more limited changes in CD3^+^ T-cell abundance or mtROS signal from those cells.

### 2.3. Higher Minimum SpO_2_ Is Associated with Better Processing Speed Performance in NP

**Figure 2:**
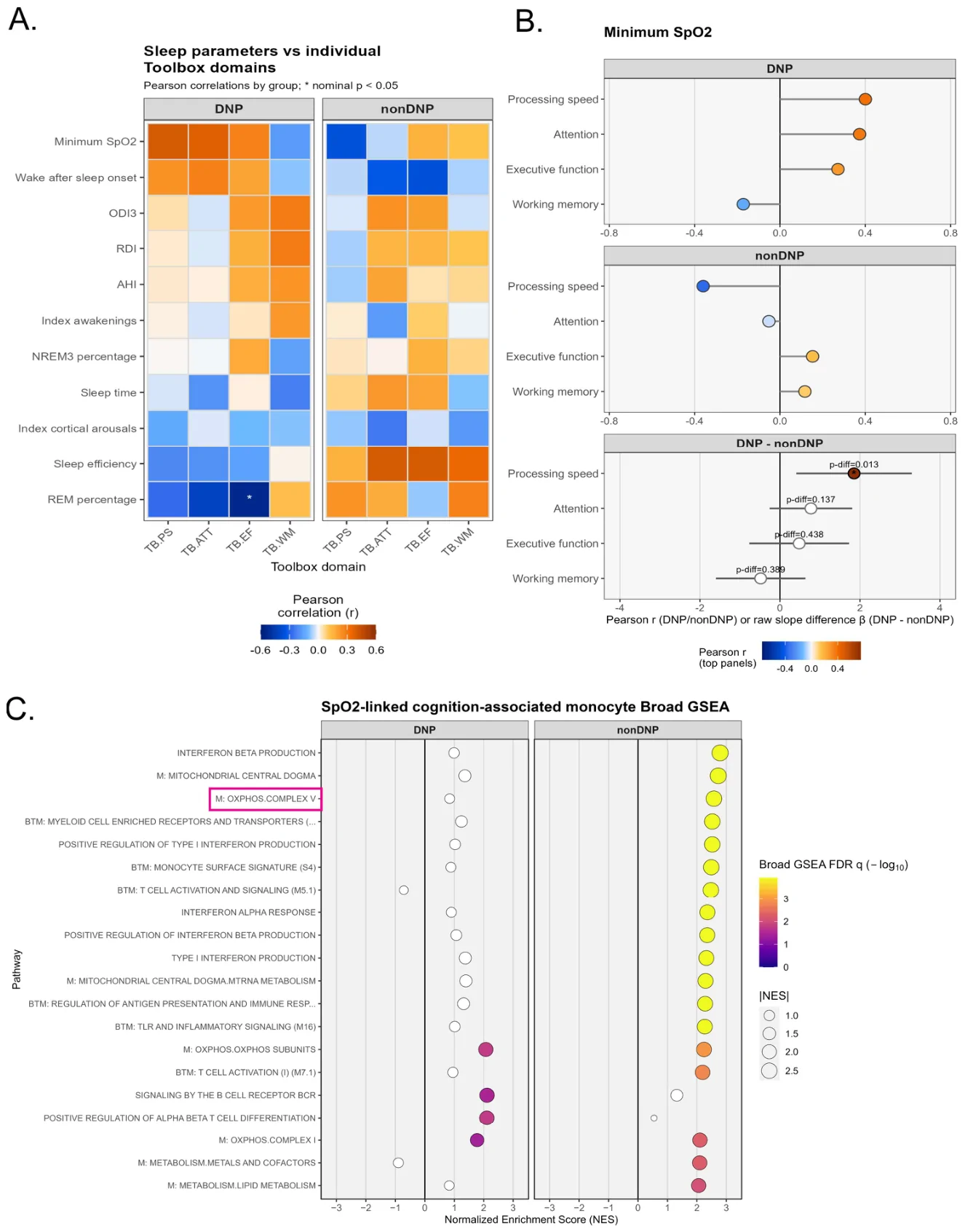
Sleep parameters associated with cognition and monocyte transcriptional programs in DNP and nonDNP participants. (**A**) Pearson correlations between Sleep Profiler parameters and individual NIH Toolbox cognitive domains. Color indicates Pearson correlation coefficient, and asterisks indicate nominal *p* < 0.05. (**B**) Associations between minimum SpO_2_ and individual NIH Toolbox cognitive domains. Point position in the DNP and nonDNP panels indicates the within-group Pearson correlation coefficient. The DNP–nonDNP panel shows the difference in unadjusted regression slopes with 95% confidence intervals; asterisks indicate a nominally significant difference in slopes (*p* < 0.05). (**C**) Monocyte GSEA after ranking genes by association with the SpO_2_-linked cognition within each group for participants with matched scRNA-seq, Sleep Profiler, and NIH Toolbox data. Point position indicates NES, point size indicates absolute NES, and point color indicates adjusted *p* value; white points indicate adjusted *p* ≥ 0.05. The red box highlights the MitoCarta Complex V pathway selected for downstream analyses. Truncated pathway labels: BTM: myeloid cell enriched receptors and transporters (M4.3); BTM: regulation of antigen presentation and immune response (M5.0). AHI: apnea-hypopnea index; ODI3: 3% oxygen desaturation index; RDI: respiratory disturbance index; NREM3: stage N3 non-rapid eye movement sleep; REM: rapid eye movement; SpO2: peripheral oxygen saturation; M: MitoCarta; BTM: blood transcription module; GSEA: gene set enrichment analysis; NES: normalized enrichment score; OXPHOS: oxidative phosphorylation.

To further characterize the relationship between sleep physiology and cognitive performance, we first analyzed objective sleep measurements in the full DISCO cohort (*n* = 43; DNP, *n* = 20; nonDNP, *n* = 23). We examined whether objective sleep physiology was associated with cognitive performance in the full DISCO cohort (*n* = 43; Table 1). Despite larger group differences in subjective measures of cognition, we prioritized NIH Toolbox scores for downstream transcriptional analyses because they provide standardized, performance-based measures of cognition. NIH Toolbox processing speed, attention, executive function, and working memory scores were analyzed separately. Pearson correlations between each sleep parameter and individual NIH Toolbox cognitive domains were calculated separately within the DNP and nonDNP groups (Figure 2A). Among DNP participants, REM sleep percentage was negatively correlated with executive function (r = −0.53, *p* = 0.016). Minimum SpO_2_ was positively correlated with processing speed in DNP participants (r = 0.40, *p* = 0.080) and negatively correlated with processing speed in nonDNP participants (r = −0.36, *p* = 0.100); the unadjusted regression slopes differed significantly between groups (*p* = 0.013) (Figure 2B). The sleep efficiency–attention slopes also differed between groups (*p* = 0.038; LOO slope difference = 1.59–2.27, *p* = 0.0037–0.0489); corresponding plots for the remaining sleep parameters are shown in Figure S1.

We next examined the 11-participant scRNA-seq subset of the DISCO cohort (DNP, *n* = 5; nonDNP, *n* = 6) to identify immune transcriptional programs associated with sleep physiology and cognitive performance. We identified immune transcriptional programs associated with sleep physiology and processing speed by analyzing scRNA-seq data using reference-based cell annotation. Unsupervised dimensional reduction was strongly influenced by donor-specific variability, therefore cells were annotated using SingleR with the MonacoImmuneData immune reference atlas. CD3^+^ T-cell and non-CD3-expressing immune cell populations were analyzed separately, and CD3^+^ T cells underwent additional reference-based annotation to obtain finer subtype labels. Downstream analyses were restricted to immune lineages represented by at least 50 cells in at least three donors from both groups. Annotated cells per participant are shown in Figure S2.

Since minimum SpO_2_ showed a group-specific association with processing speed, we investigated whether minimum SpO_2_ was associated with monocyte transcriptional programs. Within each group, monocyte genes were ranked by their association with minimum SpO_2_ for gene set enrichment analysis (GSEA). This analysis identified group-specific SpO_2_-associated transcriptional programs. In nonDNP participants, minimum SpO_2_ was strongly associated with OXPHOS-related transcriptional pathways, including ATP synthase/Complex V (Complex V). In DNP participants, this association with the Complex V transcriptional pathway was not observed, despite enrichment of other immune and mitochondrial annotations (Figure 2C).

### 2.4. Complex V Gene Expression Shows an Opposite Cognition-Associated Relationship in DNP and nonDNP Monocytes

**Figure 3:**
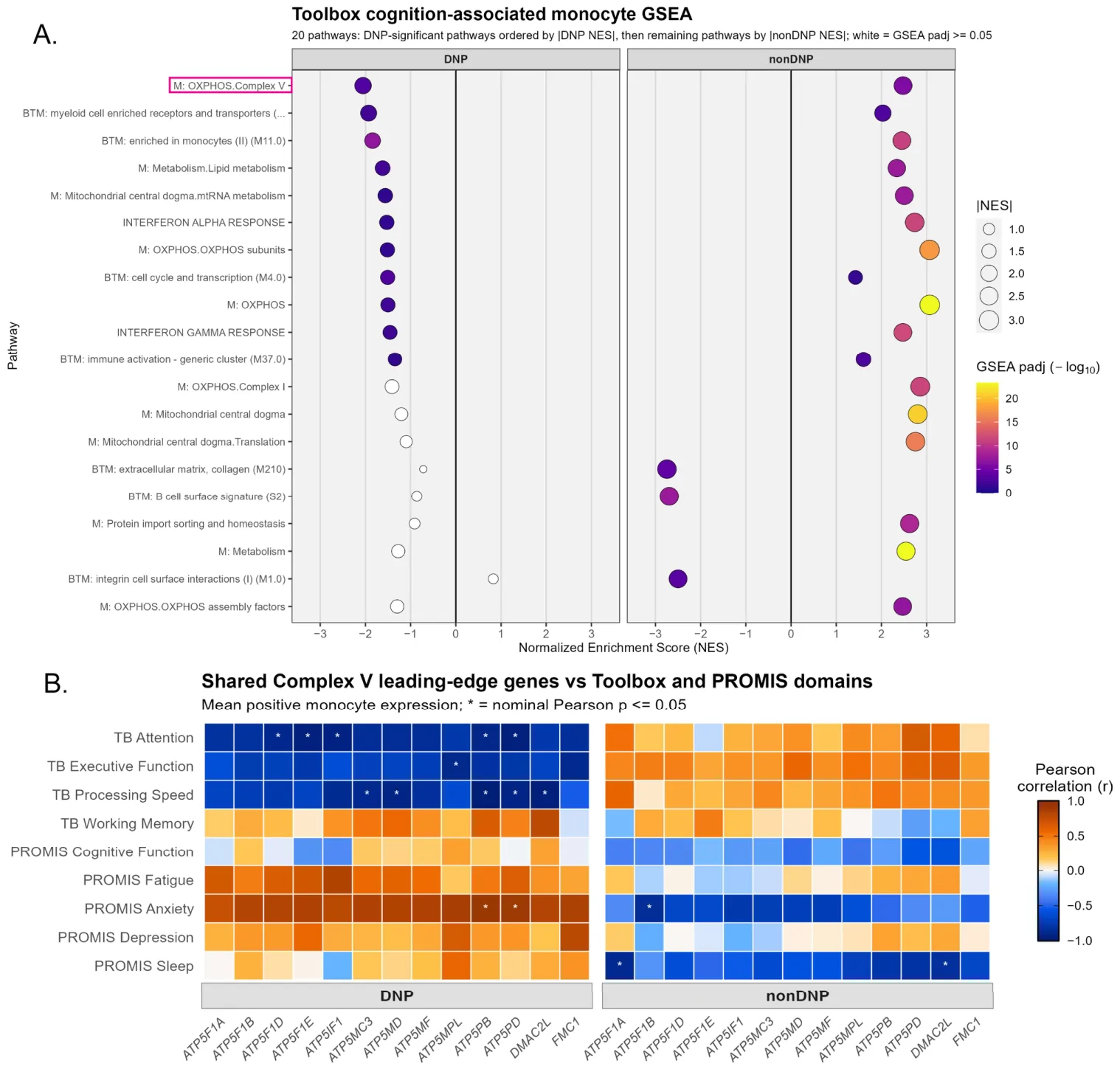
Monocyte Complex V shows opposite cognition-associated relationships in DNP and nonDNP participants. (**A**) GSEA results for selected (top 20 by DNP NES) monocyte pathways after ranking genes by association with objective cognition across NIH Toolbox domains. Point position indicates NES, point size indicates absolute NES, and point color indicates adjusted *p* value. The red box highlights the MitoCarta Complex V pathway selected for downstream analyses. White points indicate pathways that did not meet the indicated FDR threshold. (**B**) Heatmap of Pearson correlation coefficients between mean-positive monocyte expression of shared Complex V leading-edge genes and NIH Toolbox and PROMIS domain scores in DNP and nonDNP participants. Asterisks indicate Pearson *p* < 0.05. Truncated pathway label: BTM: myeloid cell enriched receptors and transporters (M4.3). M: MitoCarta; BTM: blood transcription module; H: Hallmark; GOBP: Gene Ontology Biological Process; GSEA: gene set enrichment analysis; NES: normalized enrichment score; FDR: false discovery rate; PROMIS: patient-reported outcomes measurement information system; OXPHOS: oxidative phosphorylation; TB: Toolbox; ATT: attention; EF: executive function; PS: processing speed; WM: working memory.

Within the 11-participant scRNA-seq cohort, we next identified monocyte gene programs associated with cognitive performance. We modeled NIH Toolbox performance as described above as the phenotype and participant-level monocyte mean-positive gene expression as the input. Associations were evaluated using linear mixed-effects models, with attention, executive function, processing speed, and working memory scores included as repeated domain-level outcomes, cognitive domain included as a fixed effect, and participant included as a random intercept. Genes were ranked by their association with cognitive performance using the signed t statistic for gene expression and evaluated by GSEA (Figure 3A). Pseudobulk screening identified one monocyte profile, DNP10, that exceeded the robust Mahalanobis distance screening threshold; this sample was retained as there were no abnormalities in donor technical quality control (QC) metrics (robust Mahalanobis d^2^ = 73.20, cutoff = 9.35; 0/5 technical-QC metrics flagged).

GSEA of cognition-ranked genes identified significant enrichment of multiple OXPHOS-related transcriptional pathways in DNP monocytes, with higher Complex V gene expression in monocytes associated with lower cognitive performance. Complex V was positively enriched along the SpO_2_-cognition axis in nonDNP subjects but absent from the corresponding DNP sleep-cognition analysis; we therefore asked whether the absence of this transcriptional association reflected a differing relationship between Complex V expression and cognition in NP. We compared the leading-edge genes from the nonDNP SpO_2_-linked cognition Complex V signal with those from the DNP cognition-associated Complex V signal. Thirteen genes were shared between the nonDNP SpO_2_-linked cognition Complex V leading edge and the DNP cognition-associated Complex V leading edge (*ATP5F1A*, *ATP5F1B*, *ATP5F1D*, *ATP5F1E*, *ATP5IF1*, *ATP5MC3*, *ATP5MD*, *ATP5MF*, *ATP5MPL*, *ATP5PB*, *ATP5PD*, *DMAC2L*, and *FMC1*; complete leading-edge lists for both GSEA analyses available as Table S1). These genes were designated the “monocyte anchor” because they comprised the overlapping Complex V leading-edge genes identified in the SpO_2_-linked processing speed and cognition-associated monocyte analyses, and therefore represented the monocyte phenotype associated with changes in cognition in DNP. Subject-level anchor scores were then calculated from the mean-positive expression of these genes in monocytes for use in subsequent analyses.

We additionally examined the Complex V anchor genes against the individual objective Toolbox and subjective PROMIS domains. Pearson correlations (Figure 3B) between mean-positive monocyte expression of the 13 overlapping Complex V leading-edge genes showed a coherent DNP-enriched pattern, with anchor genes generally tracking worse cognitive domain performance and higher PROMIS symptom burden, particularly anxiety-related measures. In contrast, nonDNP participants showed weaker and less consistent domain-level relationships, with some associations involving lower PROMIS symptom scores consistent with an NP-associated relationship between monocyte Complex V transcription and the cognitive burden experienced in NP. Monocyte anchor z-scores were highly stable to individual participant omission (LOO Spearman ρ = 1.00 for all omissions; minimum Pearson r > 0.9998; maximum absolute score change = 0.181).

### 2.5. Monocyte Complex V Gene Expression Correlates with Altered Coordination with CD3^+^ T-Cell Transcriptional Programs

**Figure 4:**
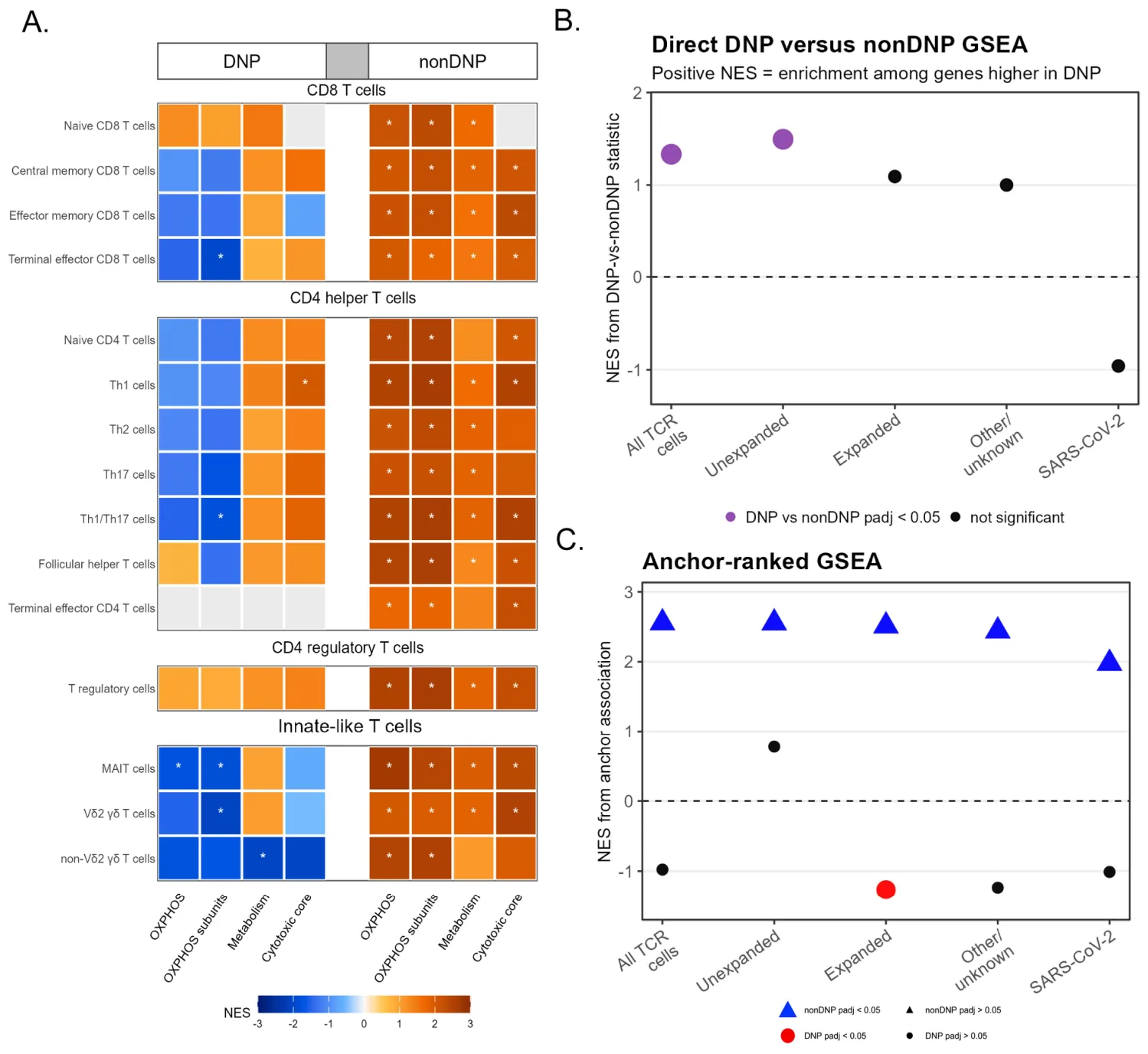
The monocyte Complex V anchor is linked to divergent CD3^+^ T-cell transcriptional programs in DNP and nonDNP participants. (**A**) Heatmap of CD3^+^ T-cell lineage-specific GSEA normalized enrichment scores (NES) for OXPHOS, OXPHOS subunits, cytotoxicity, and IFNg response after ranking genes by association with the monocyte Complex V anchor within each group. Asterisks indicate adjusted *p* < 0.05. (**B**) TcR-stratified DNP versus nonDNP GSEA for OXPHOS. Positive NES indicates gene enrichment higher in DNP. (**C**) TcR-stratified GSEA performed within each group after ranking genes by association with the monocyte OXPHOS anchor. Significant DNP enrichments are shown in red, nonDNP enrichments are shown in blue, nonsignificant enrichments in black, and gray points indicate bins with fewer than four subjects. Larger points indicate adjusted *p* < 0.05.

Within the same 11-participant scRNA-seq cohort, we then investigated whether the cognition-associated monocyte phenotype was accompanied by altered transcriptional programs in CD3^+^ T cells. The monocyte anchor score was therefore used as a continuous variable to evaluate CD3^+^ T-cell gene-expression patterns across participants. Within each CD3^+^ T-cell subset, genes were ranked by their association with the monocyte anchor separately in DNP and nonDNP participants, followed by GSEA. In nonDNP participants, increasing monocyte anchor scores were associated with broadly positive enrichment of OXPHOS, OXPHOS subunit, core cytotoxic gene expression, and interferon gamma (IFNg) programs across CD3^+^ T-cell lineages (Figure 4A). In DNP participants, however, these relationships were less consistent and varied across pathways as well as CD3^+^ T-cell subsets, with weaker or negative OXPHOS enrichment in several lineages. These findings indicate that the monocyte anchor tracked with coordinated OXPHOS- and effector-related transcriptional programs in CD3^+^ T cells in nonDNP participants, whereas this cross-compartment relationship was altered or lost in DNP. No participant-level multivariate outliers were identified in the T-cell pseudobulk profiles (0/11 participants flagged; maximum robust Mahalanobis d^2^ = 5.02, cutoff = 7.38).

To determine whether this altered monocyte–lymphocyte relationship could be attributed to adaptive immune responses established during acute SARS-CoV-2 infection as compared to a pan-lymphocytic resting state, we integrated T-cell receptor (TcR) annotations with the CD3^+^ T-cell single-cell data. Direct DNP vs. nonDNP GSEA, without accounting for the monocyte anchor, showed greater OXPHOS enrichment in DNP T cells (Figure 4B). In contrast, when genes were ranked by their association with the monocyte anchor within each group, nonDNP participants showed coordinated positive OXPHOS enrichment of CD3^+^ T cells, whereas DNP participants showed little or inconsistent T-cell OXPHOS enrichment (Figure 4C). This pattern was observed in both expanded and unexpanded T-cell populations, indicating that the altered monocyte–lymphocyte relationship in DNP was not restricted to clonally expanded or predicted antigen-specific cells.

### 2.6. Flow Cytometry Supports Altered Monocyte–Lymphocyte Mitochondrial Coordination in NP

**Figure 5:**
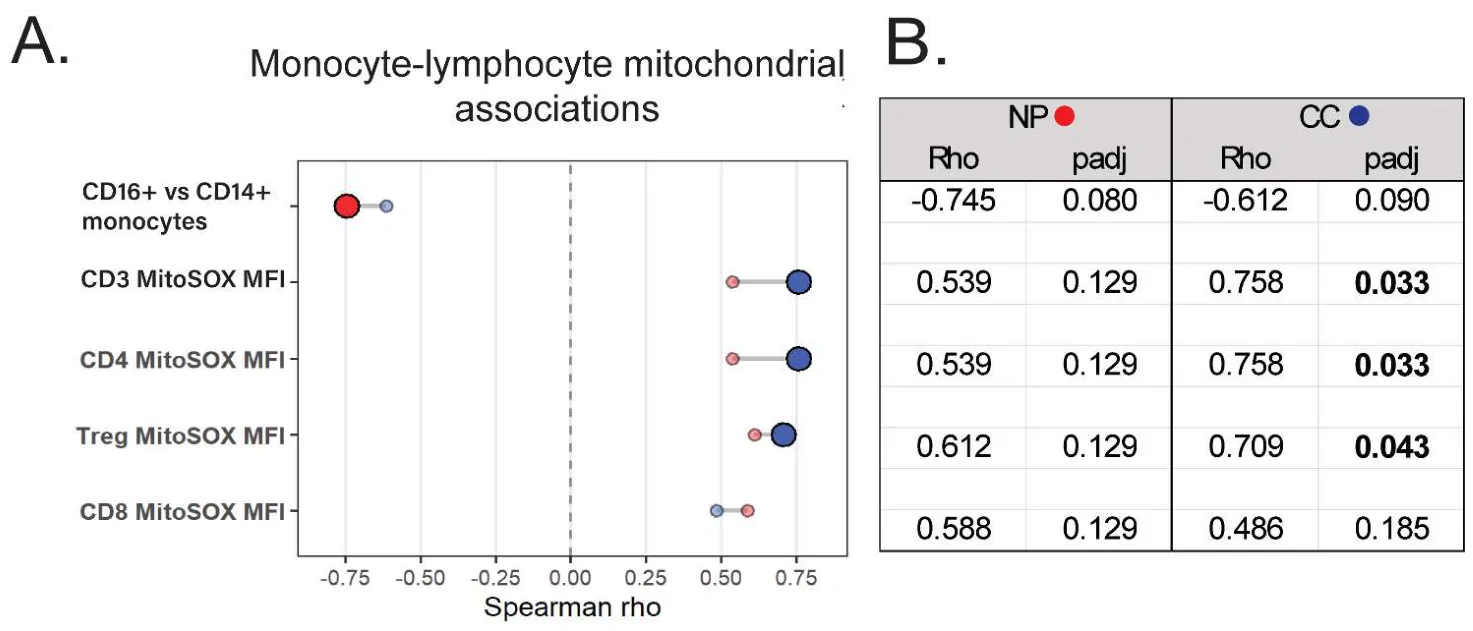
Monocyte–lymphocyte mitochondrial associations differ between CCand NP. (**A**) Spearman correlations between monocyte MitoSOX mean fluorescence intensity (MFI) and the indicated flow cytometric features in NP (red) and CC (blue) participants. Positive monocyte vs. lymphocyte mitochondrial associations with CD3, CD4, and Treg MitoSOX MFI were stronger in CC, whereas the inverse association with CD16 MitoSOX MFI within the myeloid compartment was stronger in NP. The CD8 association was weaker in both groups. Dashed line at rho = 0. (**B**) Summary table of Spearman ρ and adjusted *p* values for panel (**A**). Bold values and large circles indicate padj < 0.05. padj: Benjamini-Hochberg adjusted *p* value.

Finally, we asked whether the altered monocyte–lymphocyte OXPHOS-related transcriptional relationships observed in the scRNA-seq analyses were also reflected in the independent flow cytometry cohort. Spearman correlation analysis of monocyte MitoSOX MFI from lymphocyte and myeloid lineages showed significant positive associations between monocyte mtROS and CD3, CD4, and Treg MitoSOX signal in CC participants, which were absent in NP participants (Figure 5A,B). In contrast, the inverse association between CD16^+^ monocyte mtROS and CD14^+^ mtROS frequency within the myeloid compartment was stronger in NP, while associations with CD8 MitoSOX were weaker in both groups. These findings support a model in which NP cognitive changes are characterized by increased monocyte mitochondrial stress and an altered mitochondrial transcriptional association between monocytes and lymphocyte compartments.

## 3. Discussion

In this study, we identified a monocyte-centered mitochondrial phenotype and reduced coordination between monocyte and lymphocyte mitochondrial programs in adults over the age of 55 with NP. FC showed reduced circulating monocyte abundance together with increased monocyte mtROS in participants with NP. Among DNP participants, minimum SpO_2_ was positively associated with objective cognitive performance. In monocytes, ATP synthase/Complex V pathways were associated with both minimum SpO_2_ and cognition, and the shared leading-edge genes from these analyses defined a 13-gene monocyte anchor. Increasing anchor scores were accompanied by broadly positive OXPHOS enrichment across CD3^+^ T-cell populations in nonDNP participants, whereas this relationship was weaker or absent in DNP. The same pattern was observed in expanded and unexpanded T-cell populations, and FC correlations provided orthogonal support for reduced coordination between monocyte and lymphocyte mitochondrial programs in NP.

The association between minimum SpO_2_ and objective cognitive performance adds to existing evidence that sleep physiology and oxygenation remain altered in some individuals with long COVID/PASC. In a preliminary polysomnography study, adults with PASC had modestly lower average SpO_2_ after sleep onset and during REM sleep than controls, including after controlling for apnea [5]. Additional studies have identified altered sleep architecture or a higher burden of disordered sleep breathing in selected PASC populations [4]. However, direct evidence linking nocturnal oxygenation to cognitive symptoms associated with PASC remains limited to date. Trials of supplemental oxygen found no overall improvement in cognitive performance [24]. Altered cerebral perfusion and oxygenation have also been related to measures of cognition such as attention as well as fatigue associated with PASC, but while these central nervous system (CNS) measures may be related to peripheral SpO_2_, they remain distinct. Our findings therefore suggest that nighttime hypoxemia as measured by minimum SpO_2_ may prove relevant to cognitive performance in Neuro-PASC.

Our monocyte findings are consistent with growing evidence that SARS-CoV-2 infection is accompanied by substantial mitochondrial and metabolic remodeling in circulating immune cells. During acute infection, SARS-CoV-2 exposure can promote mtROS and HIF-1α-dependent glycolytic reprogramming in monocytes, supporting inflammatory cytokine production and impairing T-cell responses [7]. Circulating monocytes from patients with COVID-19 pneumonia have also shown mitochondrial effects such as reduced basal and maximal respiration, diminished spare respiratory capacity, depolarization, and abnormal morphology [8].

These acute studies establish that monocytes metabolically respond to SARS-CoV-2 infection, but the relevance to PASC remains less well understood, with studies showing inconsistent effects. Bulk PBMCs from individuals with PASC have shown increased basal, ATP-linked, maximal, and spare respiratory capacity compared with recovered and naive controls [9,10,11]. Conversely, a small study of monocytes from participants with cardiovascular symptoms of PASC identified impaired respiratory adaptation to oxidative challenge, abnormal membrane potential, and decreased mitochondrial DNA. Collectively, these works support persistent mitochondrial remodeling in PASC monocytes but do not define a single uniform state of increased or decreased mitochondrial activity.

This distinction is particularly important for interpreting the OXPHOS and related Complex V enrichment observed in our scRNA-seq analyses. Enrichment of OXPHOS transcripts does not directly measure oxygen consumption, ATP production, respiratory coupling, mitochondrial mass, substrate preference, or spare respiratory capacity. Prior studies suggest that increased oxygen consumption can coexist with abnormal membrane potential or impaired adaptation to oxidative stress [9,10,11]. Thus, the cognition-associated monocyte phenotype identified here should be directly measured before determining the functional effects of these findings. The concurrent elevation of monocyte MitoSOX fluorescence supports an altered mitochondrial redox state, but direct measurements, such as Seahorse, will be needed to determine whether the transcriptional program represents increased respiratory activity, compensatory remodeling, inefficient respiration, or another response to mitochondrial stress.

The central finding here is that the relationship between the monocyte phenotype and CD3^+^ T-cell transcriptional programs differed between groups. In nonDNP participants, increasing anchor scores were accompanied by coordinated positive enrichment of OXPHOS genes across multiple CD3^+^ T-cell subsets. In DNP, however, the corresponding relationships were weaker, inconsistent, or negative. Paired analyses of myeloid and lymphoid metabolism remain limited in PASC, but severe acute COVID-19 provides evidence that these compartments can develop distinct metabolic states within the same inflammatory environment. Thompson et al. identified metabolically abnormal T cells with mitochondrial dysfunction and susceptibility to apoptosis together with distinct myeloid suppressor populations in patients with severe COVID-19 [13]. Additionally, Siska et al. observed disease-stage-dependent abnormalities in mitochondrial substrate uptake, ROS accumulation, and related gene expression across both T-cell and myeloid populations [14].

Our findings may also reflect a broader transcriptomic signature of bioenergetic stress response in NP monocytes. Complex V, or mitochondrial ATP synthase, is the terminal oxidative phosphorylation complex responsible for ATP generation, but increased transcription of its component genes does not necessarily indicate increased ATP synthesis or more efficient respiration. One possibility is that the inverse relationship between Complex V-related transcription and cognition reflects compensatory induction of ATP synthase subunits in monocytes under energetic stress, particularly given the increased monocyte mtROS observed by FC. This interpretation is consistent with emerging evidence implicating mitochondrial dysfunction and impaired bioenergetics in PASC. Alternatively, sustained Complex V-related transcription could reflect maladaptive mitochondrial remodeling in which increased expression does not restore normal bioenergetic function, or it may represent a broader monocyte stress state associated with disease burden. However, because transcriptional induction does not necessarily indicate increased ATP production, future studies using direct measures of ATP synthesis will be needed to determine whether the Complex V alterations reflect compensatory upregulation, impaired ATP generation, maladaptive remodeling, or a broader stress response.

The results herein do not suggest that monocytes directly regulate T cell metabolism nor that the observed phenomenon reflects a direct interaction between the two compartments. We instead suggest that monocyte and lymphocyte mitochondrial programs may respond differently to a shared upstream factor, such as inflammatory mediators, altered nutrient availability, redox stress, hypoxia, or persistent immune stimulation. Nevertheless, the broadly positive association observed in nonDNP participants indicates that monocyte and lymphocyte mitochondrial programs varied together across individuals, whereas this association was diminished or absent in DNP. The distinction between pathway expression and transcriptomic coordination is also important. DNP T cells showed higher OXPHOS enrichment in direct group comparisons while failing to vary in parallel with the monocyte phenotype. Functional assays will be needed to determine how these transcriptional associations relate to mitochondrial metabolism and cellular function.

The TcR-stratified analyses further showed that the altered relationship was not limited to clonally expanded T cells. SARS-CoV-2-specific CD8^+^ T cells and characteristic TcR clonotypes can persist for at least two years following infection, including in people with PASC [25]. These findings indicate that this phenomenon is not dependent on SARS-CoV-2 specificity, and that persistence of antigen-specific T cells does not explain the PASC phenotype described here. In our study, similar alterations in the anchor relationship were observed in expanded and unexpanded T-cell populations. This argues against an explanation restricted to expanded clones but does not exclude contributions from persistent antigen, bystander activation, or antigen-specific cells not identified by the available TcR databases. Unexpanded cells should also not be interpreted as uniformly naïve, resting, or antigen-inexperienced.

The FC analysis provided orthogonal support for altered monocyte–lymphocyte mitochondrial relationships. In a separate group of participants without PASC, monocyte MitoSOX fluorescence was positively associated with MitoSOX fluorescence in total CD3, CD4, and regulatory T-cell populations, whereas these associations were attenuated in NP, though it is important to note that MitoSOX fluorescence and OXPHOS transcriptional enrichment measure different aspects of mitochondrial biology. The convergence of the FC and scRNA-seq analyses supports the broader conclusion that mitochondrial states across monocyte and lymphocyte compartments were more closely related in participants without NP than in those with persistent neurologic symptoms.

### Limitations

Several limitations should be considered. The analyses are exploratory, and the scRNA-seq cohort was small, limiting statistical precision and increasing the possibility that the identified genes and downstream pathway relationships are cohort-specific. Outlier screening and LOO analyses indicated that the 13-gene monocyte anchor was stable to individual donor omission, supporting its use as a summary measure of the monocyte transcriptional phenotype within this cohort. However, the broader finding of altered coordination between monocyte OXPHOS/Complex V transcriptional programs and T-cell transcriptional programs, and their connections to cognition, remains exploratory. Replication in larger, independent cohorts with paired monocyte and lymphocyte transcriptomic data will be important to determine the reproducibility and generalizability of this cross-compartment relationship.

The Complex V anchor was also defined from overlapping leading-edge genes in two related analyses: the nonDNP SpO_2_-linked cognition analysis and the DNP cognition-associated analysis. Because sleep oxygenation and cognition were associated in this cohort, these inputs were not fully independent. The resulting anchor should therefore be interpreted as a shared Complex V transcriptional signature related to the sleep-cognition relationship, rather than as a marker specific to either hypoxia or cognitive dysfunction alone. Independent cohorts and longitudinal studies will be needed to determine whether this monocyte program primarily reflects sleep-related oxygenation, cognitive dysfunction, or a broader process linking the two.

The CD3^+^ enrichment procedure represents an additional limitation because the non-CD3-expressing bystander cells carried through the isolation process may not fully represent the abundance or diversity of circulating monocytes in the original specimens. Although monocyte abundance was evaluated separately by FC, selection during cell isolation could have influenced which monocyte states were available for transcriptional analysis. In addition, although these monocytes were not directly targeted by the CD3 selection reagent, we cannot exclude transcriptional perturbations associated with ex vivo handling and passage through the enrichment workflow. The monocyte transcriptional findings should therefore be interpreted as representing the bystander monocyte population retained during CD3^+^ enrichment rather than an unbiased transcriptional profile of circulating monocytes. Cell annotations were reference-based because unsupervised dimensional reduction was strongly influenced by donor variability, and some finer immune states may not have been resolved. This study was also cross-sectional, preventing determination of changes over time. We therefore cannot establish whether the monocyte phenotype contributes to altered lymphocyte responses, whether lymphocyte changes influence monocytes, or whether both arise from a shared upstream process. Finally, pathway enrichment and MitoSOX fluorescence do not directly measure mitochondrial respiration, ATP production, coupling efficiency, or substrate use. Future studies of functional outputs should be considered.

Despite these limitations, the distinctive features found here identify altered relationships between myeloid and lymphoid mitochondrial programs in NP that are associated with decreased objective cognition. The persistence of this pattern across multiple CD3^+^ T-cell subsets, expanded and unexpanded T-cell populations, and an orthogonal FC analysis from a different set of patients suggests a broad alteration in coordination of mitochondria-associated transcriptional programs across myeloid and lymphoid compartments. Longitudinal studies combining objective sleep measures, cognitive assessment, paired monocyte and lymphocyte respirometry, metabolomics, and single-cell profiling will be required to determine the origin, direction, and functional consequences of this altered coordination.

## 4. Materials and Methods

### 4.1. Study Participants

Adult participants were enrolled through the Northwestern Medicine Neuro-COVID-19 Clinic and the DISCO study under Northwestern University Institutional Review Board (IRB) protocol STU00217720. Additional participants were enrolled from the Neuro-COVID-19 clinic or from the community under Northwestern University IRB protocol STU00212583. All participants provided written informed consent.

Subjects participated in one of three arms. Arm (1): DISCO participants with Neuro-PASC, designated as the DNP group, had a medically documented positive SARS-CoV-2 PCR or antigen test and persistent neurologic symptoms lasting at least 3 months following acute infection. Arm (2): DISCO participants were age- and sex-matched control subjects designated nonDNP with undocumented SARS-CoV-2 infection status and without symptoms of NP. Arm (3): NP patients who had a medically documented positive SARS-CoV-2 PCR or antigen test and persistent neurologic symptoms lasting at least 3 months following acute infection and age- and sex-matched COVID convalescent control participants, designated the CC group, who had a documented history of SARS-CoV-2 infection but no reported persistent post-acute symptoms.

Within arms 1 and 2, all 43 participants underwent sleep profiling and completed cognitive and quality-of-life assessments. Eleven of these participants (5 DNP and 6 nonDNP) were also included in the single-cell RNA-sequencing analysis. Final group sizes and participant overlap between the DISCO and flow cytometry cohorts are summarized in Table 1.

The analyses reported here were performed on previously collected data and were not blinded to participant group assignment.

### 4.2. Clinical, Cognitive, and Sleep Assessments

Participants completed the objective NIH Toolbox and subjective Patient-Reported Outcomes Measurement Information System (PROMIS) questionnaires at or near the time of blood collection [26,27,28]. NIH Toolbox domains included processing speed, attention, executive function, and working memory. PROMIS domain scores were expressed as standardized T-scores with a population mean of 50 and standard deviation of 10. Lower PROMIS cognitive-function scores indicated greater perceived cognitive difficulty, whereas higher fatigue, sleep-disturbance, anxiety, and depression scores indicated greater symptom severity.

Sleep assessments were obtained for all 43 DISCO participants as previously described [29]. Briefly, level 2 polysomnography was conducted in a all participants using the Sleep Profiler system (Advanced Brain Monitoring, Carlsbad, CA, USA). Measurements included the duration and percentage of rapid eye movement sleep and non-rapid eye movement sleep stages N1, N2, and N3; sleep latency; wake after sleep onset; sleep efficiency; total and index measures of cortical arousals; pulse rate; electroencephalographic spectral power, including delta or slow-wave activity; and respiratory events, including apneas, hypopneas, and oxygen desaturations.

### 4.3. Peripheral Blood Mononuclear Cell and Plasma Isolation

Venous blood samples were collected into sodium heparin tubes. Whole blood was layered over 15 mL of Histopaque-1077 density-gradient medium (Sigma-Aldrich, St. Louis, MO, USA) in 50 mL Leucosep tubes (Greiner Bio-One, Kremsmünster, Austria) and centrifuged at 1000× *g* for 18 min at room temperature.

The plasma fraction was collected and stored at −80 °C. PBMCs were collected from the density-gradient interface, washed twice with sterile PBS, and treated with ACK red blood cell lysis buffer (Quality Biological, Gaithersburg, MD, USA). Cells were either used immediately or viably cryopreserved in 10% DMSO in FBS until downstream analysis.

### 4.4. CD3^+^ T-Cell Enrichment and Single-Cell RNA Sequencing

Freshly collected PBMCs were counted and subjected to positive selection for CD3-expressing cells using magnetic beads (Miltenyi Biotec, Bergisch Gladbach, Germany; catalog no. 130-050-101). The enriched cell fraction was then prepared for droplet-based single-cell RNA (scRNA) sequencing using the 10x Genomics platform (Chromium X, 10x Genomics, Pleasanton, CA, USA). Libraries were sequenced on a NovaSeq X Plus sequencing system (Illumina, Inc., San Diego, CA, USA).

Although the enrichment procedure targeted CD3^+^ T cells, a reproducible population of non-CD3^+^ cells was retained in the captured cell suspensions. These cells were considered bystander cells carried through the positive-selection procedure. Non-CD3^+^ cells were not analyzed for frequency because the enrichment process precluded interpretation of their relative abundance, but were retained for transcriptomic analyses when the relevant cell lineage was represented in all sequenced participants.

Single-cell gene-expression libraries were generated using the 10x Genomics platform. Base-call files were processed using Cell Ranger version 7.1.0 (10x Genomics, Pleasanton, CA, USA). Reads were aligned to the human GRCh38 reference genome using GENCODE release 32/Ensembl release 98 annotations. The resulting gene-by-cell count matrices were imported into Seurat for quality control and downstream analyses.

### 4.5. Flow Cytometry

Cryopreserved PBMCs were thawed and counted, and 1 × 10^6^ cells per sample were stained with MitoSOX Red mitochondrial superoxide indicator (Invitrogen, Thermo Fisher Scientific, Waltham, MA, USA) at 37 °C according to the manufacturer’s instructions. Cells were washed and stained with viability dye (Invitrogen, Thermo Fisher Scientific) at room temperature in the dark. Following an additional wash, cells were stained with surface antibodies at 4 °C. Antibodies used for immunophenotyping are shown in Table 2.

Samples were acquired on a BD FACSymphony A1 flow cytometer (BD Biosciences, San Jose, CA, USA), with 10,000 cellular events collected per sample. Data were analyzed using FlowJo version 10 (BD Biosciences, San Jose, CA, USA). Staining and analysis were performed as previously described [30].

### 4.6. scRNA-Seq Data Processing and Quality Control

scRNA-seq data were analyzed in R version 4.4.1 using RStudio version 2024.12.1 Build 563 (Posit Software, PBC, Boston, MA, USA) on Windows. Single-cell analyses were performed using Seurat version 5.0.2 and SeuratObject version 5.1.0.

Cell Ranger filtered gene-by-cell count matrices from each participant were imported using Read10X and converted to individual Seurat objects. During initial object creation, genes detected in fewer than three cells within an individual participant and cells containing fewer than 200 detected genes were excluded. Participant-level objects were then merged into a single dataset.

The percentage of transcripts derived from mitochondrial genes was calculated for each cell. Cells were retained for analysis when they contained more than 500 and fewer than 6000 detected genes and less than 20% mitochondrial transcripts. Following quality-control filtering, gene-expression counts were normalized using Seurat’s log-normalization procedure.

### 4.7. Cell-Type Annotation and Lineage Selection

Final cell identities were assigned using a label-first strategy because unsupervised clustering approaches were dominated by interdonor variability and did not reproducibly resolve shared biologically meaningful populations across participants. Cell-level reference annotations were generated using SingleR version 2.6.0 with MonacoImmuneData from celldex version 1.14.0 as the reference dataset. Broad immune cell identities were assigned using the main Monaco reference labels, and T-cell populations were subsequently annotated using the fine-level reference labels. Reference-based annotations were evaluated using canonical lineage-marker expression and were manually reviewed and refined when supported by concordant marker patterns.

The annotated dataset was divided into T-cell and non-CD3^+^ compartments for downstream analysis. For lineage-specific analyses, only populations meeting the prespecified minimum cell-number requirements in both the DNP and nonDNP groups were retained. Final annotated objects were saved separately for the two compartments.

### 4.8. Associations of Monocyte Transcriptional Programs with Sleep and Cognitive Measures

Associations between monocyte gene expression and minimum SpO_2_ or cognitive performance were evaluated in the 11 participants included in the scRNA-seq cohort. Monocytes were identified using the broad SingleR annotation in the Seurat object. For each participant and gene, mean-positive expression was calculated as the mean normalized expression among monocytes with expression greater than zero; a value of zero was assigned when no monocytes from a participant expressed the gene.

For the minimum SpO_2_ analysis, within each group, participant-level monocyte mean-positive gene expression was modeled as a function of minimum SpO_2_ for each gene. Genes were ranked by the signed t statistic for minimum SpO_2_ and used for gene set enrichment analysis.

For the direct cognition analysis, NIH Toolbox processing speed, attention, executive function, and working memory scores were combined in a repeated-measures analysis. Associations between participant-level monocyte mean-positive gene expression and NIH Toolbox scores were calculated separately within each group using linear mixed-effects models with Toolbox domain as a fixed effect and participant ID as a random intercept.

For both analyses, genes were ranked by the corresponding within-group association statistic, and GSEA was performed separately in the DNP and nonDNP groups. The complete ranked gene universe was tested against the pathway collection contained in immune_t2g.rds. The complete gene membership for each pathway used in the analysis is provided in Supplementary Table S2.

### 4.9. Monocyte Anchor Score

For each of the 13 anchor genes, a z-score was calculated from participant-level mean-positive expression using the standard formula:

$$z=\frac{x-\mu }{\sigma }$$

where *x* is the subject mean-positive expression value for each respective anchor gene, *μ* is the mean expression of that gene across the 11 scRNA-seq participants, and *σ* is the corresponding standard deviation. The monocyte anchor score for each subject was calculated as the average of the 13 anchor-gene z-scores.

### 4.10. CD3^+^ T-Cell Subset Differential Expression and Pathway Analyses

CD3^+^ T cells were analyzed using SingleR fine annotations. For each CD3^+^ T-cell subset, raw counts were summed by subject to generate pseudobulk count matrices. Only subsets represented by at least 50 cells per subject in at least three subjects within each group were included. Genes with at least 10 counts in at least two subjects were retained. Pseudobulk counts were converted to log_2_(counts per million [CPM] + 1), and Pearson correlations between gene expression and the monocyte anchor z-score were calculated separately within the DNP and nonDNP groups.

For pathway analysis, genes were ranked by their Pearson correlation coefficient with the monocyte anchor z-score. GSEA was performed using fgsea version 1.30.0 against the immune pathway collection contained in immune_t2g.rds. Pathways were evaluated using the multilevel GSEA algorithm with the lower *p* value boundary set to zero. Benjamini–Hochberg-adjusted *p* values were calculated across all pathways tested within each group and CD3^+^ T-cell subset.

### 4.11. TcR-Stratified Anchor-Ranked Pathway Analysis

TcR clonotype annotations were assigned using paired T-cell receptor beta variable gene (TRBV) and complementarity-determining region 3 beta (CDR3β) sequences. A TcR matching key was generated for each cell as TRBV:CDR3β. Cells lacking a valid matching key were excluded from the TcR analysis. Clonotype specificity annotations were generated by comparing TRBV and CDR3β sequences with VDJdb (release 30 July 2025; https://vdjdb.cdr3.net), McPAS-TcR (http://friedmanlab.weizmann.ac.il/McPAS-TCR/, accessed on 13 August 2026), and ImmuneCODE MIRA (https://clients.adaptivebiotech.com/pub/covid-2020, accessed on 13 August 2026) entries using exact matches and near matches with a maximum Hamming or Levenshtein distance of one. Clonotypes were classified as SARS-CoV-2, autoreactive, both, or other/unknown based on the combined database annotations.

Clonotypes were considered expanded when the same TRBV:CDR3β combination was represented by at least two cells within a subject. Cells were evaluated in three analysis schemes: all cells in the TcR analysis universe; a primary expansion scheme separating unexpanded and expanded cells; and a detailed specificity scheme separating expanded other/unknown, expanded SARS-CoV-2, expanded autoreactive, and expanded clonotypes annotated as both SARS-CoV-2 and autoreactive.

For each analysis bin, raw counts were summed across cells within each subject to generate pseudobulk count matrices. Subject-level pseudobulk bins containing at least five cells were retained, and analyses were performed for group/bin combinations represented by at least three subjects. Pseudobulk counts were converted to log_2_(CPM + 1). Within each analysis bin, Pearson correlations between gene expression and the monocyte anchor z-score were calculated separately in the DNP and nonDNP groups, and genes were ranked by the resulting correlation coefficient.

NES and adjusted *p* values were determined by GSEA using the multilevel fgsea algorithm for the MITOCARTA_OXPHOS and CUSTOM_CYTOTOX_CORE pathways contained in immune_t2g.rds. Pathways with adjusted *p* < 0.05 were considered significant.

### 4.12. Associations Between Monocyte Mitochondrial Superoxide and Immune Cell Measures

Associations between monocyte mitochondrial superoxide and immune cell phenotypes were evaluated separately in the CC and NP groups. Monocyte MitoSOX fluorescence intensity was correlated with the percentages of CD14-positive and CD16-positive cells within the myeloid compartment and with MitoSOX fluorescence intensity in total CD3^+^, CD4^+^, and CD8^+^ populations as well as Tregs. Spearman correlation coefficients and corresponding *p* values were calculated using subjects with complete values for each comparison. Benjamini–Hochberg correction was applied across the tested correlations separately within each group.

### 4.13. Statistical Analysis

Statistical tests were selected according to the structure of each analysis. Two-group flow cytometry comparisons were evaluated by Mann–Whitney U tests and analyses incorporating group and cell subset factors by two-way ANOVA. Associations between continuous clinical measures were evaluated using Pearson or Spearman correlations as specified, and group differences in continuous associations were tested using regression interaction terms. Repeated NIH Toolbox cognitive domains were analyzed using linear mixed-effects models with participant as a random intercept. Ranked transcriptomic associations were evaluated by GSEA with multiple-testing correction as described in the corresponding analysis sections. Analyses were exploratory, and no a priori sample-size or power calculation was performed for the hypotheses evaluated in this study.

Outlier sensitivity analyses were performed to assess influential observations. Multivariate participant data were evaluated by PCA followed by robust Mahalanobis distance screening in the principal component space. For scRNA-seq data, participant-level pseudobulk profiles were evaluated together with donor technical quality control metrics of cell number, pseudobulk library size, median RNA count, median detected features, and median mitochondrial read percentage. Flow cytometry comparisons, minimum SpO_2_-to-processing speed interaction, and monocyte anchor z-scores were additionally assessed by LOO analysis.

### 4.14. Software and Code Availability

Software packages and applications used for data processing, statistical analysis, pathway analysis, and figure generation included GraphPad Prism version 10.4.1, Seurat version 5.0.2, SeuratObject version 5.1.0, SingleR version 2.6.0, celldex version 1.14.0, SingleCellExperiment version 1.26.0, clustree version 0.5.1, Harmony version 1.2.3, DESeq2 version 1.44.0, edgeR version 4.2.2, limma version 3.60.6, fgsea version 1.30.0, GSVA version 1.52.3, msigdbr version 25.1.1, org.Hs.eg.db version 3.19.1, Matrix version 1.7.0, dplyr version 1.1.4, tidyr version 1.3.1, readr version 2.1.5, ggplot2 version 3.5.2, pheatmap version 1.0.13, ComplexHeatmap version 2.20.0, openxlsx version 4.2.8, and vegan version 2.7.1.

## Figures and Tables

**Table 1:** Demographics.

	**Flow Cytometry Participants**				**scRNA-seq Participants**				
	Overall	NP	CC	* p *-Value	Overall	DNP	nonDNP	* p *-Value	* p *-Value DNP vs. NP
n	19	10	9		11	5	6		
Age, years (mean (1SD))	60.9 (9.6)	64.5 (5.8)	56.9 (11.6)	0.08	63.1 (6.0)	65 (3.1)	63 (7.9)	0.61	
Gender, n (%)									
Male	9 (47.4)	5 (50)	4 (44.4)	1	6 (54.5)	3 (60)	3 (50)	1	1
Female	10 (52.6)	5 (50)	5 (55.6)		5 (45.5)	2 (40)	3 (50)		
Race, n (%)									
White/Caucasian	15 (78.9)	7 (70)	8 (88.9)	1	9 (81.8)	5 (100)	4 (66.7)	0.455	1
Black or African American	1 (5.3)	1 (10)	0 (0)		2 (18.2)	0 (0)	2 (33.3)		
Multiracial	1 (5.3)	1 (10)	0 (0)						
Other	1 (5.3)	0 (0)	1 (11.1)						
Not specified *	1 (5.3)	1 (10)	0 (0)						
Ethnicity, n (%)									
Not Hispanic or Latino	18 (94.7)	9 (90)	9 (100)	1	10 (90.9)	5 (100)	4 (66.7)	1	1
Hispanic or Latino	0 (0)	0 (0)	0 (0)		1 (9.1)	0 (0)	1 (16.7)		
Not Specified *	1 (5.3)	1 (10)	0 (0)				1 (16.7)		
Toolbox									
Attention		45 (37–50)			50 (41–51)	42 (36.5–50.5)	50.5 (46–57)	0.100	0.806
Processing Speed		47 (40–56)			59 (40–66)	40 (33–64.5)	61.5 (50–66)	0.273	0.668
Executive Function		43 (39–61)			66 (47–73)	58 (46.5–68.5)	69.5 (66–73)	0.361	0.198
Working Memory		50 (44–53)			54 (50–67)	53 (49.5–62.5)	56 (50–71)	0.583	0.126
PROMIS									
Cognition		36.7 (29–43.9)			40.1 (34.2–47.4)	34.2 (27.8–35)	46.6 (41.5–48.9)	**0.006**	0.306
Anxiety		57.9 (53.9–67)			57.7 (51.2–64)	64 (56–69.3)	51.2 (46–57.7)	**0.028**	0.341
Depression		58.1 (50.5–63.9)			56 (46.1–64)	58.9 (54.5–66.5)	46.9 (34.2–57.5)	0.100	0.421
Fatigue		65.5 (59.8–69.5)			57 (47.3–73.9)	73.9 (61.6–77.3)	48.7 (39.1–57.0)	**0.011**	0.272
Sleep Disturbance		57.5 (47.3–65)			54.3 (49.2–60)	60 (53.6–68.6)	53.9 (46.3–54.3)	**0.045**	0.558
MoCA					27 (26–28)	28 (26–28.5)	26.5 (26–28)	0.315	

* Not Specified indicates that race or ethnicity information was not available in the source study records used for this analysis. Bold values indicate *p* < 0.05. Gray squares indicate that measurements were not collected. NP: NeuroPASC; CC-COVID Convalescent; scRNA-seq: single-cell RNA sequencing; DNP: DISCO Neuro-PASC: nonDNP- DISCO non-NeuroPASC; SD: Stamdard Deviation; PROMIS: Patient-Reported Outcomes Measurement Information System; MoCA: Montreal Cognitive Assessment.

**Table 2:** Antibodies used for flow cytometric immunophenotyping.

Marker	Clone	Fluorophore
CD3	OKT3	PE-Cy7
CD4	SK3	PerCP-Cy5.5
CD8	SK1	APC-Cy7
CD16	3G8	BV786
CD14	M5E2	BV711
CD127	HIL-7R-M21	BV421
CD25	M-A251	FITC

## Data Availability

Raw data supporting the findings of this study are not publicly available due to participant privacy considerations. Data may be made available from the corresponding author upon reasonable request to qualified researchers.

## Supplementary Materials

The following supporting information can be downloaded at: https://www.mdpi.com/article/10.3390/ijms27167474/s1.
