# Supplementary material for: Cognition-Linked Monocyte State Reveals Altered Myeloid–Lymphoid Coordination in Neuro-PASC
Source: Int J Mol Sci. 2026 Aug 21;27(16):7474. doi: 10.3390/ijms27167474 (PMC13513257; doi:10.3390/ijms27167474)
Supplement: Supplementary file 1 [file ijms-27-07474-s001.zip › ijms-4483503-supplementary.pdf]

Table S1: OXPHOS Complex V Leading edge genes for SpO2 and Toolbox cognition

nonDNP SpO2	Overlap	DNP Toolbox
ATP5F1C	ATP5F1A	ATP5F1C
ATP5ME	ATP5F1B	ATP5MG
ATP5MG	ATP5F1D	ATP5PF
ATP5PF	ATP5F1E	ATP5PO
ATP5PO	ATP5IF1	
ATPAF1	ATP5MC3	
MT-ATP8	ATP5MD	
	ATP5MF	
	ATP5MPL	
	ATP5PB	
	ATP5PD	
	DMAC2L	
	FMC1	

Figure S1: Sleep parameter associations with Toolbox domains

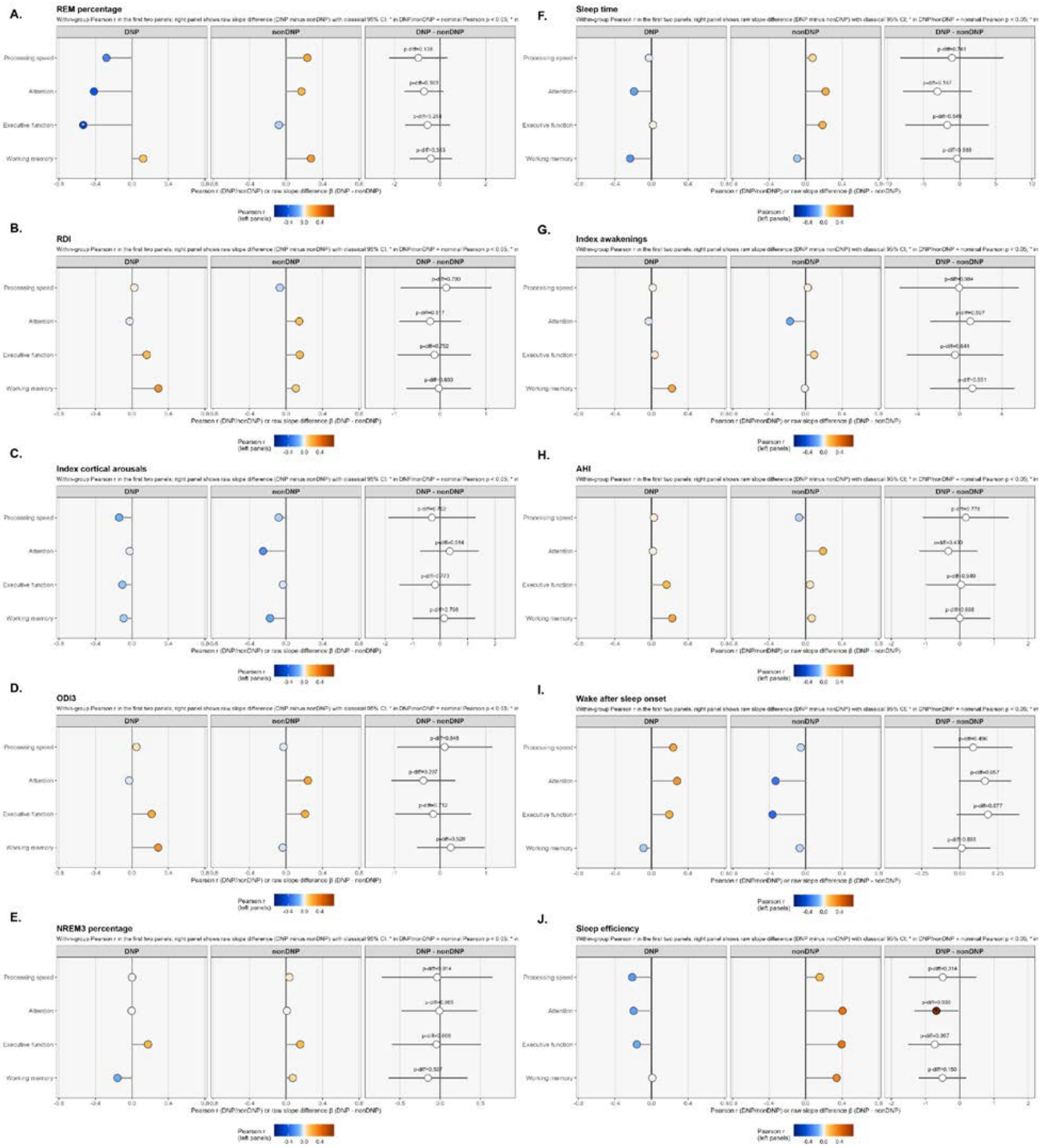

Associations of cognitive performance with (A) REM percentage, (B) respiratory disturbance index (RDI), (C) cortical arousal index, (D) 3% oxygen desaturation index (ODI3), (E) NREM3 percentage, (F) sleep time, (G) awakening index, (H) apnea–hypopnea index (AHI), (I) wake after sleep onset, and (J) sleep efficiency. Left panels show within-group Pearson correlation coefficients (P < 0.05 indicated by *), and right panels show the difference in

regression slopes between DNP and nonDNP groups (DNP − nonDNP) with 95% confidence intervals and corresponding P values for processing speed, attention, executive function, and working memory.

Figure S2: Percent of mitochondrial DNA in Annotated cells per participant

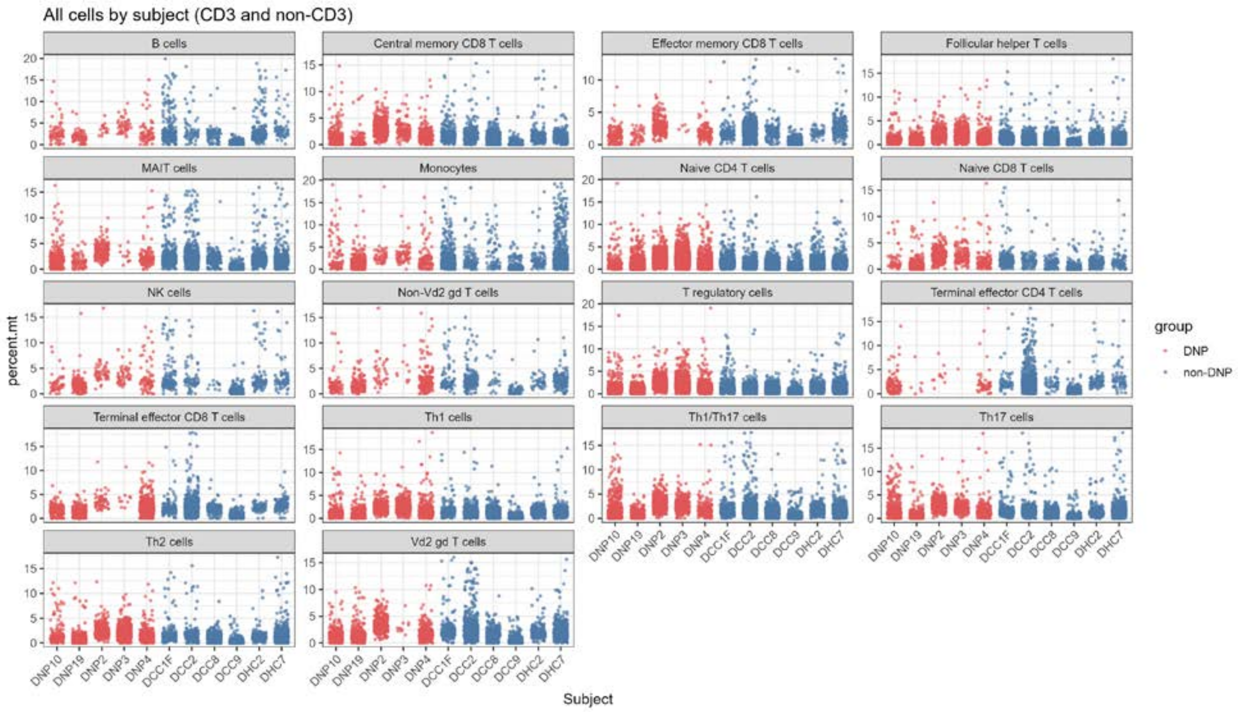

Percentage of transcripts derived from mitochondrial genes (percent.mt) is shown for individual cells from each participant, stratified by annotated cell type. Points are colored by group, with DNP participants shown in red and nonDNP participants shown in blue.

Table S2: immune_t2g.rds summmary

immune_t2g.rds summary

Metric	Value
Unique pathways	312
Unique pathway–gene memberships	2388<br>1
Unique genes	5899

Pathway	Uniq<br>ue<br>genes
BTM_Activated (LPS) dendritic cell surface signature (S11)	37

BTM_amino acid metabolishm and transport (M154.0)	33
BTM_B cell surface signature (S2)	169
BTM_cell adhesion (M51)	38
BTM_cell cycle (I) (M4.1)	145
BTM_cell cycle (III) (M103)	51
BTM_cell cycle and transcription (M4.0)	335
BTM_cell division (stimulated CD4+ T cells) (M46)	28
BTM_chemokine cluster (I) (M27.0)	26
BTM_collagen, TGFB family et al (M77)	35
BTM_DC surface signature (S5)	82
BTM_E2F1 targets (Q3) (M10.0)	33
BTM_enriched in activated dendritic cells (II) (M165)	35
BTM_enriched in B cells (I) (M47.0)	53
BTM_enriched in B cells (II) (M47.1)	43
BTM_enriched in B cells (III) (M47.2)	37
BTM_enriched in monocytes (II) (M11.0)	189
BTM_enriched in monocytes (IV) (M118.0)	57
BTM_enriched in myeloid cells and monocytes (M81)	35
BTM_enriched in neutrophils (I) (M37.1)	52
BTM_enriched in NK cells (I) (M7.2)	49
BTM_enriched in T cells (I) (M7.0)	62
BTM_extracellular matrix (I) (M2.0)	30
BTM_extracellular matrix (II) (M2.1)	45
BTM_extracellular matrix, collagen (M210)	32
BTM_immune activation - generic cluster (M37.0)	347
BTM_integrin cell surface interactions (I) (M1.0)	29
BTM_leukocyte migration (M88.0)	51
BTM_Memory B cell surface signature (S9)	37
BTM_mismatch repair (I) (M22.0)	27
BTM_mitotic cell cycle - DNA replication (M4.4)	30
BTM_mitotic cell cycle in stimulated CD4 T cells (M4.5)	35
BTM_mitotic cell division (M6)	32
BTM_Monocyte surface signature (S4)	94
BTM_myeloid cell enriched receptors and transporters (M4.3)	31
BTM_Naive B cell surface signature (S8)	54
BTM_NK cell surface signature (S1)	48
BTM_plasma cells & B cells, immunoglobulins (M156.0)	43
BTM_plasma cells, immunoglobulins (M156.1)	36
BTM_PLK1 signaling events (M4.2)	34
BTM_regulation of antigen presentation and immune response (M5.0)	81
BTM_regulation of signal transduction (M3)	47
BTM_Resting dendritic cell surface signature (S10)	75
BTM_T & B cell development, activation (M62.0)	44
BTM_T cell activation (I) (M7.1)	50
BTM_T cell activation (II) (M7.3)	31

BTM_T cell activation and signaling (M5.1)	25
BTM_T cell surface signature (S0)	27
BTM_TBA (M114.0)	39
BTM_TBA (M141)	27
BTM_TBA (M177.0)	34
BTM_TBA (M26.0)	30
BTM_TBA (source: B cells) (M152.0)	25
BTM_TLR and inflammatory signaling (M16)	45
BTM_transcription regulation in cell development (M49)	47
CELLAGE_ALL_ANTI_SENESCENCE	265
CELLAGE_ALL_PRO_SENESCENCE	229
CELLAGE_ONCOGENE_INDUCES	123
CELLAGE_ONCOGENE_INHIBITS	107
CELLAGE_REPLICATIVE_INDUCES	63
CELLAGE_REPLICATIVE_INHIBITS	87
CELLAGE_STRESS_INDUCES	80
CELLAGE_STRESS_INHIBITS	104
CUSTOM_CYTOTOX_CORE	22
CUSTOM_MHCII_CORE	25
CUSTOM_TEX_CORE	21
CUSTOM_TREG_CORE	25
CUSTOM_TYPEI_ISG_CORE	25
GOBP_ACTIVATED_T_CELL_PROLIFERATION	51
GOBP_ACUTE_INFLAMMATORY_RESPONSE	112
GOBP_ACUTE_INFLAMMATORY_RESPONSE_TO_ANTIGENIC_STIMULUS	32
GOBP_ALPHA_BETA_T_CELL_ACTIVATION	204
GOBP_ALPHA_BETA_T_CELL_DIFFERENTIATION	140
GOBP_ALPHA_BETA_T_CELL_PROLIFERATION	47
GOBP_ANTIGEN_PROCESSING_AND_PRESENTATION	120
GOBP_ANTIGEN_PROCESSING_AND_PRESENTATION_OF_ENDOGENOUS_ANTIGEN	31
GOBP_ANTIGEN_PROCESSING_AND_PRESENTATION_OF_EXOGENOUS_ANTIGEN	50
GOBP_ANTIGEN_PROCESSING_AND_PRESENTATION_OF_EXOGENOUS_PEPTIDE_ANTIGEN	41
GOBP_ANTIGEN_PROCESSING_AND_PRESENTATION_OF_EXOGENOUS_PEPTIDE_ANTIGEN_VIA_MHC_CLASS_II	31
GOBP_ANTIGEN_PROCESSING_AND_PRESENTATION_OF_PEPTIDE_ANTIGEN	72
GOBP_ANTIGEN_PROCESSING_AND_PRESENTATION_OF_PEPTIDE_ANTIGEN_VIA_MHC_CLASS_I	37
GOBP_ANTIGEN_PROCESSING_AND_PRESENTATION_OF_PEPTIDE_OR_POLYSACCHARIDE_ANTIGEN_VIA_MHC_CLASS_II	37
GOBP_B_CELL_ACTIVATION	292
GOBP_B_CELL_ACTIVATION_INVOLVED_IN_IMMUNE_RESPONSE	109
GOBP_B_CELL_APOPTOTIC_PROCESS	28
GOBP_B_CELL_DIFFERENTIATION	149
GOBP_B_CELL_HOMEOSTASIS	37
GOBP_B_CELL_MEDIATED_IMMUNITY	198
GOBP_B_CELL_PROLIFERATION	93

GOBP_B_CELL_RECEPTOR_SIGNALING_PATHWAY	78
GOBP_BROWN_FAT_CELL_DIFFERENTIATION	53
GOBP_CALCIUM_DEPENDENT_CELL_CELL_ADHESION_VIA_PLASMA_MEMBRANE_CELL_ADHESION_MOLECULES	48
GOBP_CD4_POSITIVE_ALPHA_BETA_T_CELL_ACTIVATION	135
GOBP_CD4_POSITIVE_ALPHA_BETA_T_CELL_DIFFERENTIATION	108
GOBP_CD4_POSITIVE_OR_CD8_POSITIVE_ALPHA_BETA_T_CELL_LINEAGE_COMMITMENT	36
GOBP_CD8_POSITIVE_ALPHA_BETA_T_CELL_ACTIVATION	28
GOBP_CELL_CHEMOTAXIS	328
GOBP_CELLULAR_RESPONSE_TO_INTERFERON_BETA	29
GOBP_CELLULAR_RESPONSE_TO_TYPE_II_INTERFERON	102
GOBP_CILIUM_OR_FLAGELLUM_DEPENDENT_CELL_MOTILITY	224
GOBP_CYTOKINE_PRODUCTION_INVOLVED_IN_INFLAMMATORY_RESPONSE	89
GOBP_DENDRITIC_CELL_CHEMOTAXIS	26
GOBP_ENDOTHELIAL_CELL_CHEMOTAXIS	33
GOBP_FAT_CELL_DIFFERENTIATION	260
GOBP_GAMMA_DELTA_T_CELL_ACTIVATION	28
GOBP_GRANULOCYTE_CHEMOTAXIS	121
GOBP_INFLAMMATORY_RESPONSE_TO_ANTIGENIC_STIMULUS	79
GOBP_INFLAMMATORY_RESPONSE_TO_WOUNDING	30
GOBP_INTERFERON_ALPHA_PRODUCTION	32
GOBP_INTERFERON_BETA_PRODUCTION	63
GOBP_INTERFERON_MEDIATED_SIGNALING_PATHWAY	113
GOBP_LEUKOCYTE_ACTIVATION_INVOLVED_IN_INFLAMMATORY_RESPONSE	52
GOBP_LEUKOCYTE_CHEMOTAXIS	236
GOBP_LEUKOCYTE_MEDIATED_CYTOTOXICITY	155
GOBP_LYMPHOCYTE_CHEMOTAXIS	48
GOBP_MACROPHAGE_CHEMOTAXIS	43
GOBP_MAST_CELL_ACTIVATION	74
GOBP_MAST_CELL_ACTIVATION_INVOLVED_IN_IMMUNE_RESPONSE	60
GOBP_MATURE_B_CELL_DIFFERENTIATION	42
GOBP_MONOCYTE_CHEMOTAXIS	55
GOBP_MYELOID_LEUKOCYTE_ACTIVATION	258
GOBP_NATURAL_KILLER_CELL_ACTIVATION	108
GOBP_NATURAL_KILLER_CELL_ACTIVATION_INVOLVED_IN_IMMUNE_RESPONSE	31
GOBP_NATURAL_KILLER_CELL_DIFFERENTIATION	27
GOBP_NATURAL_KILLER_CELL_MEDIATED_IMMUNITY	90
GOBP_NEGATIVE_CHEMOTAXIS	45
GOBP_NEGATIVE_REGULATION_OF_ALPHA_BETA_T_CELL_ACTIVATION	50
GOBP_NEGATIVE_REGULATION_OF_ALPHA_BETA_T_CELL_DIFFERENTIATION	30
GOBP_NEGATIVE_REGULATION_OF_B_CELL_ACTIVATION	34
GOBP_NEGATIVE_REGULATION_OF_CD4_POSITIVE_ALPHA_BETA_T_CELL_ACTIVATION	37
GOBP_NEGATIVE_REGULATION_OF_CD4_POSITIVE_ALPHA_BETA_T_CELL_DIFFERENTIATION	25
GOBP_NEGATIVE_REGULATION_OF_CHEMOTAXIS	52

GOBP_NEGATIVE_REGULATION_OF_CYTOKINE_PRODUCTION_INVOLVED_IN_INFLAMMATORY_RESPONSE	51
GOBP_NEGATIVE_REGULATION_OF_FAT_CELL_DIFFERENTIATION	62
GOBP_NEGATIVE_REGULATION_OF_INFLAMMATORY_RESPONSE	208
GOBP_NEGATIVE_REGULATION_OF_INFLAMMATORY_RESPONSE_TO_ANTIGENIC_STIMULUS	34
GOBP_NEGATIVE_REGULATION_OF_NATURAL_KILLER_CELL_MEDIATED_IMMUNITY	26
GOBP_NEGATIVE_REGULATION_OF_T_CELL_APOPTOTIC_PROCESS	25
GOBP_NEGATIVE_REGULATION_OF_T_CELL_DIFFERENTIATION	54
GOBP_NEGATIVE_REGULATION_OF_T_CELL_MEDIATED_IMMUNITY	33
GOBP_NEGATIVE_REGULATION_OF_T_CELL_PROLIFERATION	79
GOBP_NEGATIVE_REGULATION_OF_T_CELL_RECEPTOR_SIGNALING_PATHWAY	30
GOBP_NEGATIVE_REGULATION_OF_TYPE_I_INTERFERON_PRODUCTION	48
GOBP_NEGATIVE_REGULATION_OF_TYPE_II_INTERFERON_PRODUCTION	44
GOBP_NEURAL_CREST_CELL_DIFFERENTIATION	96
GOBP_NEUROINFLAMMATORY_RESPONSE	87
GOBP_NEUTROPHIL_CHEMOTAXIS	83
GOBP_PIGMENT_CELL_DIFFERENTIATION	35
GOBP_POSITIVE_CHEMOTAXIS	72
GOBP_POSITIVE_REGULATION_OF_ACTIVATED_T_CELL_PROLIFERATION	28
GOBP_POSITIVE_REGULATION_OF_ACUTE_INFLAMMATORY_RESPONSE	28
GOBP_POSITIVE_REGULATION_OF_ALPHA_BETA_T_CELL_ACTIVATION	76
GOBP_POSITIVE_REGULATION_OF_ALPHA_BETA_T_CELL_DIFFERENTIATION	57
GOBP_POSITIVE_REGULATION_OF_B_CELL_ACTIVATION	87
GOBP_POSITIVE_REGULATION_OF_B_CELL_MEDIATED_IMMUNITY	43
GOBP_POSITIVE_REGULATION_OF_B_CELL_PROLIFERATION	47
GOBP_POSITIVE_REGULATION_OF_CD4_POSITIVE_ALPHA_BETA_T_CELL_ACTIVATION	49
GOBP_POSITIVE_REGULATION_OF_CD4_POSITIVE_ALPHA_BETA_T_CELL_DIFFERENTIATION	39
GOBP_POSITIVE_REGULATION_OF_CHEMOTAXIS	145
GOBP_POSITIVE_REGULATION_OF_CYTOKINE_PRODUCTION_INVOLVED_IN_INFLAMMATORY_RESPONSE	30
GOBP_POSITIVE_REGULATION_OF_FAT_CELL_DIFFERENTIATION	72
GOBP_POSITIVE_REGULATION_OF_GRANULOCYTE_CHEMOTAXIS	27
GOBP_POSITIVE_REGULATION_OF_INFLAMMATORY_RESPONSE	166
GOBP_POSITIVE_REGULATION_OF_INTERFERON_ALPHA_PRODUCTION	26
GOBP_POSITIVE_REGULATION_OF_INTERFERON_BETA_PRODUCTION	42
GOBP_POSITIVE_REGULATION_OF_LEUKOCYTE_CHEMOTAXIS	97
GOBP_POSITIVE_REGULATION_OF_NATURAL_KILLER_CELL_ACTIVATION	26
GOBP_POSITIVE_REGULATION_OF_NATURAL_KILLER_CELL_MEDIATED_CYTOTOXICITY	30
GOBP_POSITIVE_REGULATION_OF_NATURAL_KILLER_CELL_MEDIATED_IMMUNITY	36
GOBP_POSITIVE_REGULATION_OF_SUBSTRATE_ADHESION_DEPENDENT_CELL_SPREADING	43
GOBP_POSITIVE_REGULATION_OF_T_CELL_CYTOKINE_PRODUCTION	28
GOBP_POSITIVE_REGULATION_OF_T_CELL_MEDIATED_CYTOTOXICITY	38
GOBP_POSITIVE_REGULATION_OF_T_CELL_MEDIATED_IMMUNITY	72
GOBP_POSITIVE_REGULATION_OF_T_CELL_MIGRATION	37

GOBP_POSITIVE_REGULATION_OF_T_CELL_PROLIFERATION	108
GOBP_POSITIVE_REGULATION_OF_TYPE_I_INTERFERON_PRODUCTION	77
GOBP_POSITIVE_REGULATION_OF_TYPE_II_INTERFERON_PRODUCTION	78
GOBP_POSITIVE_T_CELL_SELECTION	51
GOBP_PRODUCTION_OF_MOLECULAR_MEDIATOR_INVOLVED_IN_INFLAMMATORY_RESPONSE	121
GOBP_PYROPTOTIC_INFLAMMATORY_RESPONSE	40
GOBP_REGULATION_OF_ACUTE_INFLAMMATORY_RESPONSE	49
GOBP_REGULATION_OF_ALPHA_BETA_T_CELL_ACTIVATION	124
GOBP_REGULATION_OF_ALPHA_BETA_T_CELL_DIFFERENTIATION	82
GOBP_REGULATION_OF_B_CELL_ACTIVATION	135
GOBP_REGULATION_OF_B_CELL_DIFFERENTIATION	37
GOBP_REGULATION_OF_B_CELL_MEDIATED_IMMUNITY	62
GOBP_REGULATION_OF_B_CELL_PROLIFERATION	68
GOBP_REGULATION_OF_B_CELL_RECEPTOR_SIGNALING_PATHWAY	25
GOBP_REGULATION_OF_BROWN_FAT_CELL_DIFFERENTIATION	25
GOBP_REGULATION_OF_CD4_POSITIVE_ALPHA_BETA_T_CELL_ACTIVATION	86
GOBP_REGULATION_OF_CD4_POSITIVE_ALPHA_BETA_T_CELL_DIFFERENTIATION	64
GOBP_REGULATION_OF_CHEMOTAXIS	215
GOBP_REGULATION_OF_CILIUM_DEPENDENT_CELL_MOTILITY	32
GOBP_REGULATION_OF_FAT_CELL_DIFFERENTIATION	148
GOBP_REGULATION_OF_GRANULOCYTE_CHEMOTAXIS	53
GOBP_REGULATION_OF_INFLAMMATORY_RESPONSE	451
GOBP_REGULATION_OF_INFLAMMATORY_RESPONSE_TO_ANTIGENIC_STIMULUS	49
GOBP_REGULATION_OF_LEUKOCYTE_CHEMOTAXIS	126
GOBP_REGULATION_OF_LYMPHOCYTE_CHEMOTAXIS	25
GOBP_REGULATION_OF_MACROPHAGE_CHEMOTAXIS	30
GOBP_REGULATION_OF_MAST_CELL_DEGRANULATION	29
GOBP_REGULATION_OF_MONOCYTE_CHEMOTAXIS	32
GOBP_REGULATION_OF_NATURAL_KILLER_CELL_ACTIVATION	45
GOBP_REGULATION_OF_NATURAL_KILLER_CELL_MEDIATED_IMMUNITY	59
GOBP_REGULATION_OF_NEUROINFLAMMATORY_RESPONSE	42
GOBP_REGULATION_OF_NEUTROPHIL_CHEMOTAXIS	33
GOBP_REGULATION_OF_POSITIVE_CHEMOTAXIS	26
GOBP_REGULATION_OF_SUBSTRATE_ADHESION_DEPENDENT_CELL_SPREADING	61
GOBP_REGULATION_OF_T_CELL_ACTIVATION	401
GOBP_REGULATION_OF_T_CELL_APOPTOTIC_PROCESS	43
GOBP_REGULATION_OF_T_CELL_DIFFERENTIATION_IN_THYMUS	28
GOBP_REGULATION_OF_T_CELL_MEDIATED_CYTOTOXICITY	48
GOBP_REGULATION_OF_T_CELL_MEDIATED_IMMUNITY	107
GOBP_REGULATION_OF_T_CELL_MIGRATION	52
GOBP_REGULATION_OF_T_CELL_RECEPTOR_SIGNALING_PATHWAY	53
GOBP_REGULATION_OF_TYPE_I_INTERFERON_MEDIATED_SIGNALING_PATHWAY	45
GOBP_REGULATORY_T_CELL_DIFFERENTIATION	43
GOBP_RESPONSE_TO_INTERFERON_BETA	37

GOBP_RESPONSE_TO_TYPE_I_INTERFERON	92
GOBP_RESPONSE_TO_TYPE_II_INTERFERON	128
GOBP_SUBSTRATE_ADHESION_DEPENDENT_CELL_SPREADING	100
GOBP_SUBSTRATE_DEPENDENT_CELL_MIGRATION	29
GOBP_T_CELL_ACTIVATION_INVOLVED_IN_IMMUNE_RESPONSE	139
GOBP_T_CELL_APOPTOTIC_PROCESS	61
GOBP_T_CELL_CHEMOTAXIS	29
GOBP_T_CELL_CYTOKINE_PRODUCTION	40
GOBP_T_CELL_DIFFERENTIATION	327
GOBP_T_CELL_DIFFERENTIATION_IN_THYMUS	89
GOBP_T_CELL_DIFFERENTIATION_INVOLVED_IN_IMMUNE_RESPONSE	98
GOBP_T_CELL_HOMEOSTASIS	50
GOBP_T_CELL_LINEAGE_COMMITMENT	42
GOBP_T_CELL_MEDIATED_CYTOTOXICITY	60
GOBP_T_CELL_MEDIATED_IMMUNITY	139
GOBP_T_CELL_MIGRATION	79
GOBP_T_CELL_PROLIFERATION	225
GOBP_T_CELL_RECEPTOR_SIGNALING_PATHWAY	152
GOBP_T_CELL_SELECTION	64
GOBP_TROPHOBLAST_CELL_MIGRATION	25
GOBP_TYPE_I_INTERFERON_PRODUCTION	131
GOBP_TYPE_II_INTERFERON_MEDIATED_SIGNALING_PATHWAY	28
GOBP_TYPE_II_INTERFERON_PRODUCTION	123
GOBP_VIRAL_RELEASE_FROM_HOST_CELL	29
HALLMARK_ALLOGRAFT_REJECTION	200
HALLMARK_COMPLEMENT	200
HALLMARK_GLYCOLYSIS	200
HALLMARK_IL2_STAT5_SIGNALING	199
HALLMARK_IL6_JAK_STAT3_SIGNALING	87
HALLMARK_INFLAMMATORY_RESPONSE	200
HALLMARK_INTERFERON_ALPHA_RESPONSE	97
HALLMARK_INTERFERON_GAMMA_RESPONSE	200
HALLMARK_TNFA_SIGNALING_VIA_NFKB	200
MITOCARTA_Metabolism	461
MITOCARTA_Metabolism.Amino_acid_metabolism	90
MITOCARTA_Metabolism.Carbohydrate_metabolism	73
MITOCARTA_Metabolism.Detoxification	51
MITOCARTA_Metabolism.Electron_carriers	15
MITOCARTA_Metabolism.Lipid_metabolism	123
MITOCARTA_Metabolism.Metals_and_cofactors	122
MITOCARTA_Metabolism.Nucleotide_metabolism	40
MITOCARTA_Metabolism.Sulfur_metabolism	11
MITOCARTA_Metabolism.Vitamin_metabolism	49
MITOCARTA_Mitochondrial_central_dogma	231
MITOCARTA_Mitochondrial_central_dogma.mtDNA_maintenance	31

MITOCARTA_Mitochondrial_central_dogma.mtRNA_metabolism	76
MITOCARTA_Mitochondrial_central_dogma.Translation	155
MITOCARTA_Mitochondrial_dynamics_and_surveillance	104
MITOCARTA_Mitochondrial_dynamics_and_surveillance.Apoptosis	35
MITOCARTA_Mitochondrial_dynamics_and_surveillance.Autophagy	18
MITOCARTA_Mitochondrial_dynamics_and_surveillance.Cristae_formation	11
MITOCARTA_Mitochondrial_dynamics_and_surveillance.Fission	15
MITOCARTA_Mitochondrial_dynamics_and_surveillance.Fusion	9
MITOCARTA_Mitochondrial_dynamics_and_surveillance.Intramitochondrial_membrane_interactions	9
MITOCARTA_Mitochondrial_dynamics_and_surveillance.Mitophagy	14
MITOCARTA_Mitochondrial_dynamics_and_surveillance.Organelle_contact_sites	14
MITOCARTA_Mitochondrial_dynamics_and_surveillance.Trafficking	7
MITOCARTA_OXPHOS	169
MITOCARTA_OXPHOS.Complex_I	66
MITOCARTA_OXPHOS.Complex_II	8
MITOCARTA_OXPHOS.Complex_III	16
MITOCARTA_OXPHOS.Complex_IV	51
MITOCARTA_OXPHOS.Complex_V	26
MITOCARTA_OXPHOS.Cytochrome_C	2
MITOCARTA_OXPHOS.OXPHOS_assembly_factors	68
MITOCARTA_OXPHOS.OXPHOS_subunits	102
MITOCARTA_OXPHOS.Respirasome_assembly	4
MITOCARTA_Protein_import_sorting_and_homeostasis	86
MITOCARTA_Protein_import_sorting_and_homeostasis.Protein_homeostasis	51
MITOCARTA_Protein_import_sorting_and_homeostasis.Protein_import_and_sorting	48
MITOCARTA_Signaling	48
MITOCARTA_Signaling.Calcium_homeostasis	24
MITOCARTA_Signaling.cAMP-PKA_signaling	6
MITOCARTA_Signaling.Immune_response	5
MITOCARTA_Small_molecule_transport	85
MITOCARTA_Small_molecule_transport.ABC_transporters	8
MITOCARTA_Small_molecule_transport.Calcium_uniporter	6
MITOCARTA_Small_molecule_transport.Sideroflexins	5
MITOCARTA_Small_molecule_transport.SLC25A_family	51
REACTOME_ADORA2B_MEDIATED_ANTI_INFLAMMATORY_CYTOKINES_PRODUCTION	43
REACTOME_ANTIGEN_ACTIVATES_B_CELL_RECEPTOR_BCR_LEADING_TO_GENERATION_OF_SECOND_MESSENGERS	86
REACTOME_ANTIGEN_PRESENTATION_FOLDING_ASSEMBLY_AND_PEPTIDE_LOADING_OF_CLASS_I_MHC	29
REACTOME_CD22_MEDIATED_BCR_REGULATION	61
REACTOME_DDX58_IFIH1_MEDIATED_INDUCTION_OF_INTERFERON_ALPHA_BETA	82
REACTOME_DISEASES_OF_IMMUNE_SYSTEM	32
REACTOME_DOWNSTREAM_SIGNALING_EVENTS_OF_B_CELL_RECEPTOR_BCR	68
REACTOME_DOWNSTREAM_TCR_SIGNALING	89
REACTOME_EVASION_BY_RSV_OF_HOST_INTERFERON_RESPONSES	35

REACTOME_INTERFERON_ALPHA_BETA_SIGNALING	78
REACTOME_INTERFERON_GAMMA_SIGNALING	98
REACTOME_INTERFERON_SIGNALING	280
REACTOME_MHC_CLASS_II_ANTIGEN_PRESENTATION	126
REACTOME_SIGNALING_BY_THE_B_CELL_RECEPTOR_BCR	153
REACTOME_TCR_SIGNALING	111
